# A Novel Route Controlling Begomovirus Resistance by the Messenger RNA Surveillance Factor Pelota

**DOI:** 10.1371/journal.pgen.1005538

**Published:** 2015-10-08

**Authors:** Moshe Lapidot, Uri Karniel, Dana Gelbart, Doron Fogel, Dalia Evenor, Yaarit Kutsher, Zion Makhbash, Sahadia Nahon, Haviva Shlomo, Lea Chen, Moshe Reuveni, Ilan Levin

**Affiliations:** 1 Department of Vegetable and Field Crop Research, Institute of Plant Sciences, Agricultural Research Organization, Volcani Center, Bet Dagan, Israel; 2 Department of Ornamental Plants and Agricultural Biotechnology, Institute of Plant Sciences, Agricultural Research Organization, Volcani Center, Bet Dagan, Israel; Virginia Tech, UNITED STATES

## Abstract

*Tomato yellow leaf curl virus* (TYLCV) is a devastating disease of tomato (*Solanum lycopersicum*) that can be effectively controlled by the deployment of resistant cultivars. The TYLCV-resistant line TY172 carries a major recessive locus for TYLCV resistance, designated *ty-5*, on chromosome 4. In this study, the association between 27 polymorphic DNA markers, spanning the *ty-5* locus, and the resistance characteristics of individual plants inoculated with TYLCV in 51 segregating recombinant populations were analyzed. These analyses localized *ty-5* into a 425 bp region containing two transversions: one in the first exon of a gene encoding the tomato homolog of the messenger RNA surveillance factor Pelota (*Pelo*), and a second in its proximal promoter. Analyses of susceptible and resistant lines revealed that the relative transcript level of the gene remained unchanged, regardless of whether the plants were infected with TYLCV or not. This suggests that the polymorphism discovered in the coding region of the gene controls the resistance. Silencing of *Pelo* in a susceptible line rendered the transgenic plants highly resistant, while in the resistant line TY172 had no effect on symptom development. In addition, over-expression of the susceptible allele of the gene in the resistant TY172 line rendered it susceptible, while over-expression of the resistant allele in susceptible plants had no effect. These results confirm that *Pelo* is the gene controlling resistance at the *ty-5* locus. *Pelo*, implicated in the ribosome recycling-phase of protein synthesis, offers an alternative route to promote resistance to TYLCV and other viruses.

## Author Summary

Tomato is one of the most important food crops worldwide, providing phytonutrients and color to our diet. *Tomato yellow leaf curl virus* (TYLCV) is one of the most devastating viruses of cultivated tomatoes and a key limiting factor to tomato production in major tomato-growing areas. The management of TYLCV is difficult because its whitefly vector populations can reach high numbers and relies heavily on the application of hazardous chemicals (insecticides). Breeding TYLCV-resistant tomato cultivars provides an attractive, environmentally friendly strategy to reduce yield losses caused by the virus. Considerable efforts have been invested in breeding TYLCV-resistant tomato cultivars. The discovery and utilization of genes controlling resistance can expedite the breeding process and highlight innate means to promote such resistance. Here we report the discovery of the gene controlling TYLCV resistance at the *ty-5* locus. It was found that the messenger RNA surveillance factor Pelo is responsible for this resistance. Our discovery, together with the recent discovery of the gene controlling resistance at the *Ty-1*/*Ty-3* locus, can lead to precision pyramiding of these two genes to enhance the spectrum of resistance of tomato plants to TYLCV, and possibly to other begomoviruses.

## Introduction

*Tomato yellow leaf curl virus* (TYLCV) is one of the most devastating viruses of cultivated tomatoes, *Solanum* (*S*.) *lycopersicum*. Although first identified in the eastern Mediterranean [1], it has spread into almost all tropical and subtropical regions [2, 3]. TYLCV induces severe cupping of apical leaves, yellowing, and stunting, resulting in considerable yield losses. TYLCV has become a major limiting factor to tomato production in major tomato-growing areas, including: China, Mexico, Florida and California [4, 5].

TYLCV is a monopartite begomovirus (family *Geminiviridae*) transmitted by the whitefly *Bemisia tabaci* (Gennadius). Population outbreaks of whiteflies are often associated with a high incidence of the disease [6]. The virus genome is composed of a single circular single-stranded DNA molecule of about 2,800 nucleotides. Management of TYLCV is difficult because its whitefly vector populations can reach enormous numbers. Therefore, breeding TYLCV-resistant tomato cultivars provides an attractive, environmentally sound, strategy to reduce yield losses inflicted by the virus [4, 7–9].

Considerable efforts have been invested in breeding TYLCV-resistant tomato cultivars [4]. As all cultivated tomato accessions are susceptible to the disease, wild tomato species were screened to identify, map and introgress resistance loci into *S*. *lycopersicum*. Among these are: *Ty-2* that was introgressed from *S*. *habrochaites*, *ty-5* presumably from *S*. *peruvianum*, and *Ty-1*, *Ty-3* and *Ty-4* from *S*. *chilense* accessions [10–17]. Because *Ty-1* and *Ty-3* are allelic [18, 19], the number of available genes conferring TYLCV resistance is quite limited. Recently, the gene responsible for TYLCV-resistance at the *Ty-1*/*Ty-3* locus was identified and shown to code for an RNA-dependent RNA polymerase (RDR) [19]. Moreover, it was shown that *Ty-1*/*Ty-3* confers resistance to TYLCV by increasing cytosine methylation of the viral genome, indicating that the resistance conferred by this locus acts through viral transcriptional gene silencing [20]. Thus far, no other gene conferring TYLCV resistance has been unambiguously identified.

Line TY172, carrying *ty-5*, is thought to be derived from four different wild tomato accessions, three of *S*. *peruvianum*: PI 126926, PI 126930, PI 390681, and one of *S*. *Arcanum*: LA0441 [21, 22]. The breeding procedure yielding TY172 was described before [17, 21].

TY172 is highly resistant to TYLCV: it shows no disease symptoms following infection and contains low levels of viral DNA [23]. TY172 exhibited the highest level of resistance in a field trial that compared fruit yield of various resistant accessions following TYLCV-inoculation [23]. It was also found that TY172, probably due to its high level of TYLCV-resistance, is a poor source for viral acquisition and transmission by whiteflies [24]. These characteristics emphasize the high potential of utilizing TY172 in breeding TYLCV-resistant tomato cultivars.

Classical segregation studies suggested that resistance in TY172 is controlled by three genes exerting a partially-dominant effect [21]. However, a study designed to map genes controlling TYLCV-resistance in TY172 showed that resistance is conferred by a previously unknown major recessive quantitative trait locus (QTL), termed *ty-5*, that maps to chromosome 4 and four minor OTLs [17].

Recently, a recessive resistance carried by the old commercial cultivar Tyking (Royal Sluis, The Netherlands) has been shown to co-localize with *ty-5* [25]. The authors suggested that because one of the populations used by Anbinder et al. [17] also displayed a recessive gene action, the resistance in Tyking most likely corresponds to the resistance in TY172.

TYLCV-resistance inherited by TY172 at the *ty-5* locus is highly associated with a gene encoding a NAC **DOMAIN 1** protein (Nac1) [17]. Nac1 was previously implicated in the replication of another tomato-infecting begomovirus ToLCV, by interacting with its replication enhancer protein (REn) in cultivated tomato [26]. It was further shown that ToLCV induce *Nac1* expression in infected susceptible cells, and that this up-regulation requires REn. Also, in a transient ToLCV replication system, over-expression of *Nac1* resulted in a substantial increase in viral DNA accumulation. These results suggest that *Nac1* plays an important role in replication-enhancement of ToLCV and possibly other begomoviruses, including TYLCV, in susceptible plants. Therefore, this gene, or more precisely its homolog in TY172, was initially referred to as a candidate gene reducing TYLCV replication and thus conferring resistance at the *ty-5* locus [17].

The objective of this study was to fine-tune map *ty-5*. For this purpose, the associations between 27 polymorphic DNA markers spanning the *ty-5* locus, including *Nac1*, and the resistance characteristics of individual plants inoculated with TYLCV in segregating populations were analyzed. These analyses, coupled with transgenic confirmation, identified the gene controlling resistance at the *ty-5* locus as the tomato homolog of the messenger RNA surveillance factor *Pelo*.

## Results

### Fine-tune mapping and general characteristics of the *ty-5* locus

To fine-tune map the introgression in the TYLCV-resistant line TY172, carrying the *ty-5* gene, we have sequenced 800-to-900 bp fragments of its genome spanning the *Nac1* gene region (first, approximately every 50 Kilo bp (Kbp), then every 10 Kbp and finally every 3 Kbp and at times even in smaller intervals). These sequencing results were compared to the sequence of the reference genome Heinz 1706, known to be susceptible to TYLCV (build SL2.40 in http://solgenomics.net/) in order to identify nucleotide polymorphisms. These polymorphisms were validated by sequencing M-82 as well. From these sequence comparisons a core set of 27 markers were generated and used throughout this study (S1 Table). Genomic DNA sequence comparisons between the resistant TY172 line and its susceptible counterparts did not yield significant insertions or deletions usually characterizing DNA sequences of species distantly related to the cultivated tomato, including *S*. *peruvianum*. In addition, no polymorphism was detected upstream of the *Nac1* promoter gene-sequence beyond those displayed (S1 Fig). These results indicate that the control of TYLCV resistance is exerted either by *Nac1*, characterized by Tyrosine^212^-to-Cysteine substitution in TY172 (S2 Fig), or by a gene located downstream from its position on chromosome 4.

### *Nac1* is not the gene controlling TYLCV resistance at the *ty-5* locus

**Fig 1:**
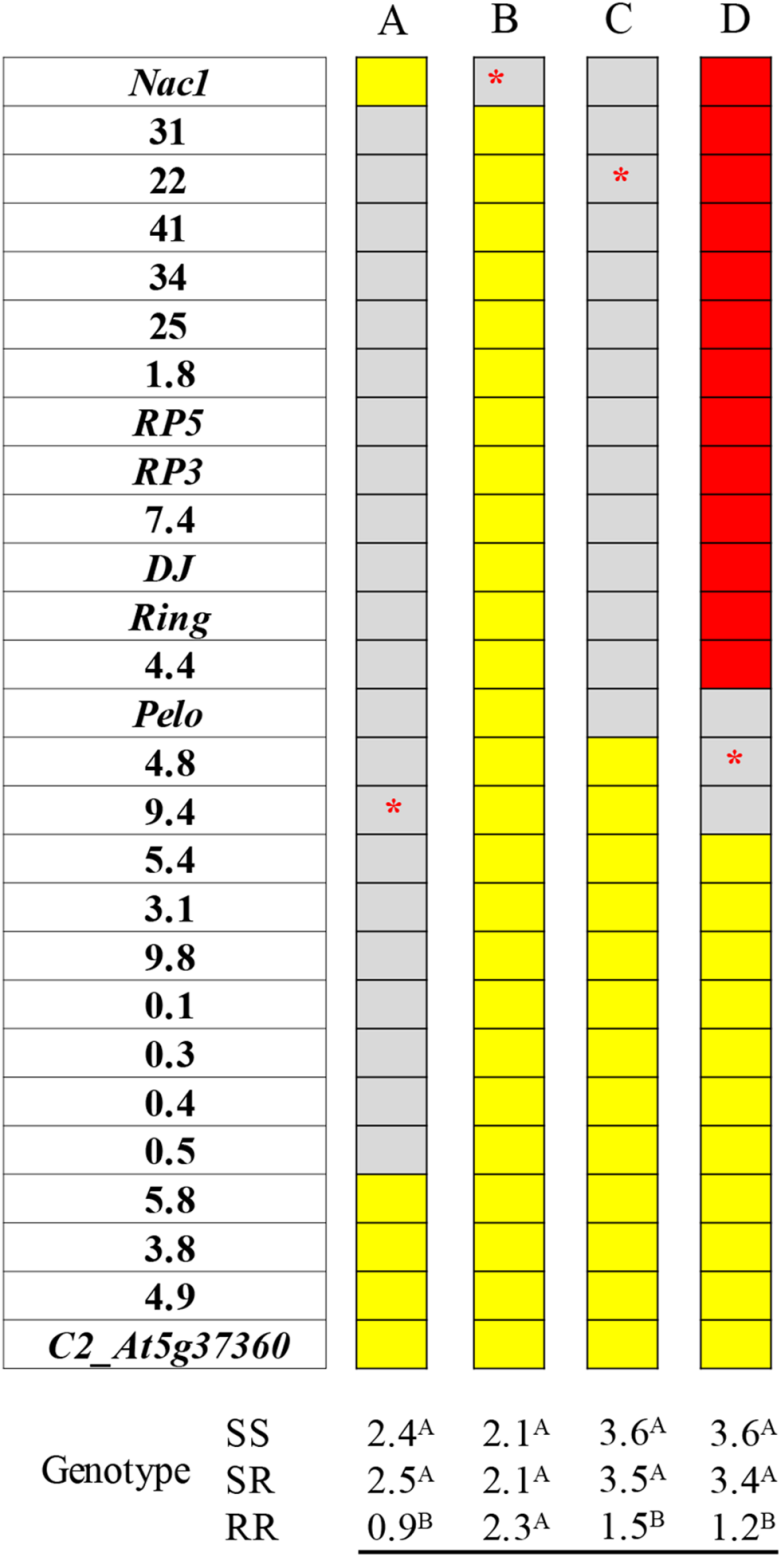
Association between DNA markers spanning the *ty-5* locus and disease severity in representative segregating populations. In the ruler presented to the left of each analysis: yellow-shaded markers are homozygous for the allele originated from the susceptible line M-82 (SS), red-shaded markers are homozygous for the allele originated from the resistant line TY172 (RR) and gray-shaded markers are heterozygous (SR); the analysis of variance presented at the bottom of each population was carried out with different markers: the marker in red asterisks is the one that was used as an independent variable in each analysis; different superscript letters above means indicate statistically significant difference, *P*<0.05, between genotypes for each population separately; A-D: four different representative segregating populations; DSI (disease severity index) was determined at 28 DPI (days post inoculation) and at 42 DPI, the DSI values presented is an average of both readings.

To carry out the map-based analysis of *ty-5*, a series of 51 recombinant lines were generated. As this analysis progressed, it became evident that *Nac1* is not the gene controlling TYLCV-resistance at the *ty-5* locus. For example, a BC_1_F_3_ population fixed for the *Nac1* allele originating from the susceptible line M-82 and segregating for markers 31 through 0.5 still displayed a strong association between the resistance and segregating markers at this range (Fig 1A). On the other hand, a BC_2_F_3_ population segregating solely for *Nac1* did not display a significant association between the gene and disease symptoms (Fig 1B). These results delimit the *ty-5* gene into approximately 351 Kb between *Nac1* and the 5.8 marker (S1 Table).

### Recombination frequency and map-based identification of the *ty-5* gene

We have analyzed 5,662 plants from different segregating populations following a cross between the resistant TY172 and the susceptible M-82 line. 51 plants displaying recombination events among the polymorphic DNA markers were identified. These plants were allowed to self-pollinate to produce segregating populations that were again genotyped, inoculated and assayed for resistance (S3 Fig). These 51 recombinant plants enabled us to narrow down the resistant locus into a 23,250 bp fragment (in TY172), including the amplicons produced by markers 4.4 and 4.8, containing two genes: a *Calcium dependent protein kinase* (*Cdpk2*) [27] and the *Pelo* gene homolog [28] (S3 Fig). The identification of this region can be exemplified as follows:

1. A BC_1_F_3_ population segregating for the region flanked by *Nac1* and marker 4.8 still displayed a strong association between the TYLCV-resistance trait and markers segregating at this range (Fig 1C). These results further delimit the *ty-5* gene into a 268.2 Kb fragment between *Nac1* and *Pelo*.
2. An F_3_ population (Fig 1D) that is: A. Fixed for the region originating from TY172 between *Nac1* and marker 4.4; B. Fixed for the region originating from M-82 between marker 5.4 and marker *C2_At5g37360*; and C. Segregating for a region flanked by markers 4.4 and 5.4, continued to display a strong association between the TYLCV-resistance trait and the segregating markers (Fig 1D). These results (Fig 1), confirmed by 47 additional recombinant populations, 32 of which are presented in S3 Fig, demonstrate that the *ty-5* gene resides between markers 4.4 and 4.8.

**Fig 2:**
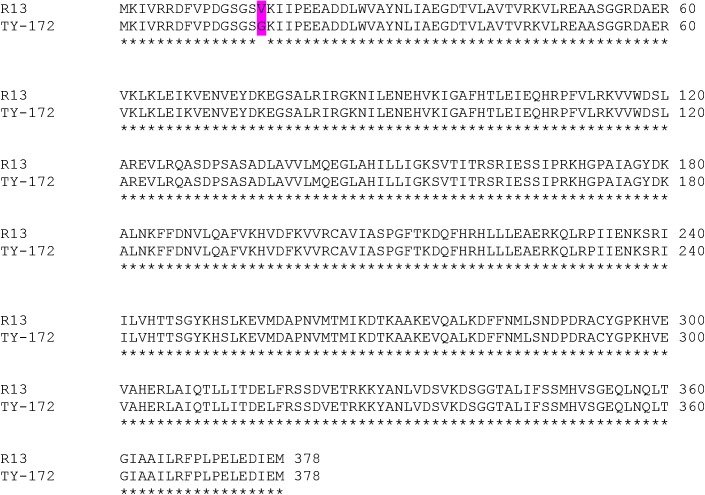
Amino-acid sequence of the *Pelo* gene in the resistant TY172 line compared to the susceptible line M-82. The substitution of Valine^16^ (susceptible lines) to a Glycine (resistant TY172 line) is highlighted with magenta; GenBank accession numbers for TY172 and M-82 are KC447285 and KC447286, respectively.

The region between markers 4.4 and 4.8 was sequenced in TY172 and M-82 and compared to the sequence of Heinz 1706. Seven single nucleotide polymorphisms (SNPs) were found in this region. Further sequencing of the polymorphic region between *Pelo* and marker 4.4 in selected recombinant plants of populations such as the one displayed in Fig 1D have shown that the three SNPs found in this region (between *Pelo* and marker 4.4, the region in which *Cdpk2* is located) are unrelated to the resistance. This clearly demonstrated that *Cdpk2* is not involved in the resistance, and enabled us to delimit the introgression into a polymorphic 2,386 bp region between *Pelo* and marker 4.8. The SNPs in this region were: a G^1^-to-A transition, single nucleotide insertion (A^1565^), and a T^1961^-to-A^1962^ transversion in the promoter region of the *Pelo* gene in TY172 (S4 Fig). In addition, a T^47^-to-G transversion in the first exon of *Pelo* was also observed (T^2385^-to-G^2386^ in S4 Fig). Additional sequencing of the polymorphic region between *Pelo* and marker 4.8 in selected recombinant plants obtained from populations such as the one displayed in Fig 1C enabled us to finally delimit the *ty-5* gene into a 425 bp region containing two transversions. One transversion is the T^1961^-to-A^1962^ in the *Pelo* proximal promoter region and the other is the T^47^-to-G in the first exon of the gene (S4 Fig), resulting in a Valine^16^-to-Glycine substitution in TY172 (Fig 2). These results show that *Pelo* is the gene controlling TYLCV resistance at the *ty-5* locus and that this resistance can be either attributed to its proximal promoter or its coding region. Noteworthy, other polymorphisms, but none of these SNPs, were found following sequencing of this region in DNA extracted from two-to-three plants each of PI 126926, PI 126930, PI 390681 and LA0441, the four accessions claimed to be the origin of *ty-5* (demonstrated by their *Pelo* coding sequence in GenBank accession numbers KC567248 through KC567257). Moreover, screening of the 360 tomato genomes provided by Lin et al 2014 [29] showed no polymorphism at the two transversions mentioned above (the T^1961^-to-A^1962^ in the *Pelo* proximal promoter and the T^47^-to-G in the first exon of the gene).

Sequence analysis of a polymorphic region tightly linked to *Pelo* displayed complete identity between two plants of the old tomato hybrid Tyking genomic DNA with TY172 DNA (S4 Fig). Moreover, the amino-acids sequence of *Pelo* from Tyking was identical to its counterpart from TY172 (Fig 2). It is therefore highly likely that *ty-5* in TY172 and Tyking originated from the same unknown source. Because to the best of our knowledge Tyking preceded TY172, we cannot exclude the possibility that *ty-5* in TY172 was introgressed from Tyking. Nonetheless, the origin of *ty-5* locus in Tyking is yet to be established.

### Transcriptional analysis of *Pelo*

To test whether the SNP in the *Pelo* promoter-region is involved in the resistant phenotype, relative transcript level of the gene was compared between resistant and susceptible genotypes. The results show that relative transcript level of *Pelo* in the resistant line TY172 was not statistically different from the susceptible line M-82, whether the plants were infected with TYLCV or not (Table 1A). The relative transcript level of *Pelo* was also analyzed in another susceptible line used in this study, R13. The relative transcript level of *Pelo* in the resistant line TY172 was not statistically different from this susceptible line as well (Table 1B). These results suggest that the SNP in the *Pelo* promoter region is not associated with TYLCV resistance.

**Table 1:** Average relative transcript levels of the *Pelo* gene in resistant TY172 and susceptible M-82 and R13 plants.

			Days post inoculation			
Experiment	Line	Treatment	7	14	21	28
A	TY172	Inoculated	1.4^A^ ± 0.3	2.7^A^ ± 0.6	3.2^A^ ± 0.6	1.1^A^ ± 0.3
		Non-inoculated	1.5^A^ ± 0.2	2.1^A^ ± 0.3	4.3^A^ ± 1.1	1.1^A^ ± 0.3
	M-82	Inoculated	1.5^A^ ± 0.1	1.1^A^ ± 0.2	3.1^A^ ± 0.7	1.1^A^ ± 0.2
		Non-inoculated	2.4^A^ ± 0.3	1.6^A^ ± 0.3	1.6^A^ ± 0.2	1.7^A^ ± 0.4
B	TY172	Inoculated	2.2^A^ ± 0.1	4.4^A^ ± 0.9	1.1^A^ ± 0.2	1.9^A^ ± 0.2
		Non-inoculated	2.1^A^ ± 0.2	2.9^A^ ± 0.4	1.1^A^ ± 0.1	2.0^A^ ± 0.3
	R13	Inoculated	1.3^A^ ± 0.4	2.3^A^ ± 0.3	1.1^A^ ± 0.2	1.4^A^ ± 0.3
		Non-inoculated	2.6^A^ ± 0.5	2.6^A^ ± 0.3	1.0^A^ ± 0.2	1.6^A^ ± 0.2

The two experiments, A and B, were performed in two different years and analyzed independently. Ten samples, each sample composed of three different plants, were analyzed for each line, treatment and date. Identical superscript letters indicate that there is no statistical difference, *P*>0.05, between means for each date separately within each experiment. ± SEM.

### Over-expression of *Pelo*

To confirm that indeed the SNP in the *Pelo* coding-region is responsible for the resistance phenotype, transgenic plants over-expressing each of the two *Pelo* alleles were developed. Two independent populations of transgenic TY172 plants segregating for pBIN*Pelo*-M-82 were analyzed (Table 2). Following inoculation with TYLCV, all transgenic plants displayed disease symptoms while their respective azygous controls were symptomless, similarly to inoculated non-transgenic TY172 plants. The transgenic plants showed an average disease severity index (DSI) values of 2.2 and 2.3 for TYT-10 and TYT-94, respectively, correlated with an approximately 3.5-fold higher average virus copy number (VCN) in transgenic plants as compared to their non-transgenic azygous controls (Table 2). The two transgenic TY172 lines were also assayed for transcript-expression levels of *Pelo* (Table 2). In both transgenic lines there was a significant increase in *Pelo* transcript level as compared to their non-transgenic azygous controls, a five-fold increase in the transgenic line TYT-10 (a T_1_ generation), and a 91-fold increase in the transgenic line TYT-94 (a T_3_ generation). These results clearly demonstrate that TY172 plants over-expressing the susceptible allele of *Pelo* are no longer resistant to TYLCV, although their DSI is lower than that expected from fully susceptible plants. A possible explanation can be the presence of the minor OTLs identified in TY172 [17].

**Table 2:** Effect of over-expressing the *Pelo* allele from M-82 plants in transgenic TY172 plants.

Line	Genotype^a^	n		Relative *Pelo* transcript level	Fold increase in *Pelo* transcript level^b^	DSI^c^	Relative VCN (X10^4^)^d^
TYT-10	NT	10		1^B^		0.1^B^±0.1	1.5^B^±0.7
	T	21		5^A^	5	2.2^A^±0.1	4.5^A^±0.6
			*P*	1X10^-4^	1X10^-4^	7X10^-11^	1X10^-2^
TYT-94	NT	10		1^B^		0.0^B^±0.0	4.4^B^±0.7
	T	13		91^A^	91	2.3^A^±0.1	14.5^A^±2.8
			*P*	2X10^-11^	2X10^-11^	2X10^-13^	6X10^-3^

^a^ NT = Not transgenic; T = Transgenic

^b^ Fold increase of *Pelo* transcription depicts the level of *Pelo* transcript level in the transgenic plants divided by the transcript level in the non-transgenic plants.

^c^ DSI = TYLCV disease severity index. Plants were scored and sampled 28 days post inoculation.

^d^ VCN = Virus copy number

Different superscript letters indicate statistically significant differences between transgenic and non-transgenic plants for each trait separately.

Three independent T_1_ populations of R13 plants segregating for pBIN*Pelo*-TY172 were also analyzed. While RNA level of the transgene was significantly increased in these lines (179, 229 and 173-fold in TYT-002, TYT-066 and TYT-103, respectively), no statistical difference was found in average DSI values between the transgenic and their azygous counterparts (Table 3). In all the three lines there was also no statistical difference in VCN between the transgenic plants and their azygous controls. These results show that over-expression of the resistant allele of *Pelo* does not affect the TYLCV-susceptible R13 plants, which is in agreement with the recessive nature of the *ty-5* locus in segregating populations (Fig 1A, 1C and 1D).

**Table 3:** Effect of over-expressing the *Pelo* allele derived from TY172 plants in transgenic R13 plants.

Line	Genotype^a^	n		Relative *Pelo* transcript level	Fold increase in *Pelo* transcript level^b^	DSI^c^	Relative VCN (X10^4^)^d^
TYT-002	NT	10		1.0^B^		3.5^A^±0.1	10.5^A^±2.2
	T	11		179.0^A^	179	3.5^A^±0.2	5.8^A^±0.5
			*P*	2X10^-9^	2X10^-9^	9X10^-1^	3X10^-1^
TYT-066	NT	10		1.0^B^		3.9^A^±0.2	9.1^A^±1.1
	T	9		229.0^A^	229	3.6^A^±0.2	6.8^A^±1.9
			*P*	1X10^-11^	1X10^-11^	2X10^-1^	4X10^-1^
TYT-103	NT	11		1.0^A^		3.8^A^±0.1	13.9^A^±3.2
	T	15		173^B^	173	4.0^A^±0.1	13.0^A^±2.1
			*P*	2X10^-12^	2X10^-12^	1X10^-1^	9X10^-1^

^a^ NT = Not transgenic; T = Transgenic

^b^ Fold increase of *Pelo* transcription depicts the level of *Pelo* transcript level in the transgenic plants divided by the transcript level in the non-transgenic plants.

^c^ DSI = TYLCV disease severity index. Plants were scored and sampled 28 days post inoculation.

^d^ VCN = Virus copy number

Different superscript letters indicate statistically significant differences between transgenic and non-transgenic plants for each trait separately.

### Silencing of *Pelo*

To further confirm that indeed the *Pelo* coding-region is responsible for the resistance phenotype, transgenic TYLCV-susceptible (R13) and resistant (TY172) plants harboring a *Pelo* silencing construct (pHannibal-*Pelo*) were developed. Three independent T_1_ populations of transgenic R13 plants segregating for the silencing construct pHannibal-*Pelo* were analyzed (Table 4). Following inoculation with TYLCV, all the transgenic plants showed no disease symptoms while their respective azygous controls showed severe disease symptoms (yellowing and cupping of the leaves, most clearly shown in the plant apex) (S5 Fig), similarly to inoculated R13 control plants (S6 Fig). These transgenic plants showed an average DSI values of practically 0, correlated with a 2.2-to-3.5 fold reduction in *Pelo* transcript level, and a 20-to-60 fold decrease in average VCN in transgenic plants as compared to their non-transgenic azygous controls (Table 4). However, when the two independent T_1_ populations of transgenic TY172 plants segregating for the silencing construct were analyzed there were no differences in disease severity between the transgenic plants and their respective azygous controls (Table 4 and S6 Fig). Both transgenic and azygous control alike showed no disease symptoms, with a DSI of 0, coupled with a six-to-nine fold reduction in *Pelo* transcript level in the transgenic plants as compared to their non-transgenic azygous controls (Table 4). Interestingly, despite the lack of difference in DSI, there was a significant difference in virus copy number: the transgenic plants showed a nine-to-tenfold decrease in VCN compared to their non-transgenic azygous control plants (Table 4).

**Table 4:** Effect of *Pelo* silencing in transgenic susceptible (R13) and resistant (TY172) plants.

Type	Line	Genotype ^a^	n		Relative *Pelo* transcript level	Fold reduction in *Pelo* transcript level ^b^	DSI ^c^	Relative VCN (X10^4^) ^d^
*Pelo* RNAi in TY172	TYT-411	NT	5		1.0^A^	5.7	0.0^A^±0.0	105^A^±24
		T	11		0.18^B^		0.0^A^±0.0	12^B^±6
				*P*	2X10^-3^	2X10^-3^	1.0	3X10^-4^
	TYT-412	NT	6		1.0^A^	9.1	0.0^A^±0.0	77^A^±30
		T	18		0.11^B^		0.0^A^±0.0	8^B^±5
				*P*	4X10^-3^	4X10^-3^	1.0	6X10^-3^
*Pelo* RNAi in R13	TYT-413	NT	13		1.0^A^	2.6	3.2^A^±0.2	307^A^±44
		T	13		0.38^B^		0.0^B^±0.0	5^B^±3
				*P*	2X10^-3^	2X10^-3^	1X10^-15^	4X10^-7^
	TYT-414	NT	8		1.0^A^	2.2	3.5^A^±0.1	562^A^±97
		T	13		0.46^B^		0.1^B^±0.1	31^B^±2
				*P*	7X10^-3^	7X10^-3^	1X10^-15^	1X10^-6^
	TYT-415	NT	10		1.0^A^	3.5	3.7^A^±0.2	563^A^±67
		T	15		0.28^B^		0.0^B^±0.0	14^B^±6
				*P*	6X10^-4^	6X10^-4^	5X10^-20^	8X10^-13^

^a^ NT = Not transgenic; T = Transgenic

^b^ Fold reduction of *Pelo* transcription depicts the level of *Pelo* transcription in the non-transgenic plants divided by the transcription level in the transgenic plants.

^c^ DSI = TYLCV disease severity index. Plants were scored and sampled 28 days post inoculation.

^d^ VCN = Virus copy number

Different superscript letters indicate statistically significant differences between transgenic and non-transgenic plants for each trait separately.

### The effect of *ty-5* in non-inoculated plants

A nearly isogenic BC_4_F_3_ segregating-population was developed by crossing TY172 (RR) as a maternal line and M-82 (SS) as a recurrent paternal line, using *Pelo* as the sole marker. Non-inoculated homozygous RR plants of this population displayed a significant reduction in fruit size, and a small insignificant reduction in total fruit yield and harvest index compared to the recurrent SS parent (Table 5). However, when compared to their homozygous SS counterparts the homozygous RR plants displayed a significant reduction of 23% in total yield and a 27% reduction in fruit size, suggesting that the presence of *ty-5* may exerts a penalty.

**Table 5:** Yield components, per plant, of segregating non-inoculated BC_4_F_3_ plants in comparison to their recurrent parent M-82.

Genotype	Total fruit weight (kg)	Av. Fruit number (n)	Av. fruit size (gr)	Plant weight (gr)	Harvest index
M-82	3.1^AB^ ± 0.1	70^A^ ± 3	45^A^ ± 1	1381^A^ ± 68	2.3^A^ ± 0.1
SS	3.4^A^ ± 0.1	72^A^ ± 3	47^A^ ± 1	1364^A^ ± 60	2.6^A^ ± 0.2
SR	3.1^AB^ ± 0.2	74^A^ ± 5	43^A^ ± 2	1341^A^ ± 77	2.4^A^ ± 0.2
RR	2.6^B^ ± 0.2	77^A^ ± 5	34^B^ ± 2	1433^A^ ± 93	2.0^A^ ± 0.2

RR = plants homozygous for *Pelo* originating from TY172; SS = plants homozygous for *Pelo* originating from M-82; SR = heterozygous plants; different superscript letters indicate statistically significant differences, *P*<0.05, between genotypes for each trait separately; ± SEM.

## Discussion

TY172 is a tomato line expressing high level TYLCV-resistance. Based on a classical segregation study, it was suggested that the resistance is controlled by three genes that originated from *S*. *peruvianum* [21]. Subsequently, molecular mapping studies showed that TYLCV-resistance in TY172 is controlled by a major recessive QTL, termed *ty-5*, and four additional minor QTLs. The major QTL maps to chromosome 4 while the minor QTLs were mapped to chromosomes 1, 7, 9 and 11 [17].

This study was designed to identify the gene controlling TYLCV-resistance at the *ty-5* locus. To accomplish this, a core set of 27 polymorphic DNA markers were designed. The use of 51 informative recombinant populations enabled us to exclude *Nac1*, a candidate gene suggested earlier [17], and delimits the *ty-5* locus into a single gene encoding the tomato homolog of the messenger RNA surveillance factor Pelo. Two SNPs were identified: one in Pelo’s proximal promoter region and the other in its coding sequence. Our results further show that the relative transcript level of *Pelo* in the resistant TY172 plants was not statistically different from susceptible plants, either inoculated or not. It can therefore be suggested that the control of TYLCV-resistance is exerted by the SNP observed in the *Pelo* coding sequence, and not by the one observed in its proximal promoter region. These two SNPs and others observed upstream to their location could not be traced in sequences of two-to-three plants of each of the four *S*. *peruvianum* accessions claimed to be the origin of *ty-5* [21]. This suggests that the *Pelo* allele residing in TY172 originated from a different source or that it represents a rare allele segregating in one or more of these four accessions. Noteworthy, *Pelo* and its upstream promoter sequence from the cultivar Tyking, carrying a recessive resistance co-localized with *ty-5* [25], were found identical to TY172. Although we cannot exclude the possibility that the resistant *Pelo* allele originated from Tyking, we were not able to trace its origin in wild and other cultivated tomato accessions, including in those of the 360 tomato genomes provided by Lin et al 2014 [29].

Over-expression of the cultivated *Pelo* allele in TY172 resulted in increased viral titer and disease symptoms, while over-expression of its resistant counterpart in susceptible plants had no effect on TYLCV titer and disease severity. In addition, silencing of *Pelo* in susceptible plants rendered these transgenic plants highly resistant to TYLCV—the inoculated plants showed practically no disease symptoms, and a 20-to-60 fold reduction in virus titer compared to non-transgenic control plants. Finally, silencing *Pelo* in resistant TY172 plants had no effect on disease severity. Nonetheless, these transgenic plants had a nine-to-tenfold decrease in virus titer compared to their non-transgenic control plants. Taken together, these results confirm that *Pelo* controls the TYLCV-resistance at the *ty-5* locus.

Pelo was recently implicated in the recycling phase of protein biosynthesis as part of eukaryotic and archaeal ribosome recycling complexes containing also an ABC-type ATPase (Abce1) [28]. Ribosome-driven protein biosynthesis has four phases: initiation, elongation, termination and recycling. Interestingly, recessive genes for resistance to plant viruses have been linked to components of the eukaryotic translation initiation complex. Translation initiation factors, particularly the eIF4E and eIF4G protein families, were found to be essential for RNA virus infections [30]. Here we show that a protein implicated in the latest phase of ribosome-driven protein biosynthesis controls the recessive resistance to TYLCV, a DNA virus.

Recessive resistance genes are more prevalent controlling resistance to viruses than resistance to fungal or bacterial pathogens which is primarily a dominant genetic trait [30]. Because typical plant viruses encode relatively few proteins, between four-to-ten, and need to recruit many different host components to complete their infection cycle, it was proposed that resistance genes would correspond to mutation or loss of host components required for a stage of the virus life cycle [31]. This fits well with the recessive nature attributed in this study to the *Pelo* allele variant residing in TY172.

Although many host factors are required for plant virus infections [32], analysis of recessive resistance identified in the natural diversity of crops has thus far only revealed a group of proteins linked to the translation machinery [30]. TYLCV-resistance controlled by the *ty-5* gene *Pelo* is not an exception, but points to the ribosome recycling phase of protein synthesis, rather than to its initiation, as an intervening step promoting resistance.

Recycling of ribosomes for a new round of translation initiation is an essential part of protein synthesis. As recently summarized [28], Abce1, mentioned above, can dissociate ribosomes into subunits either after canonical termination by release factors (Rfs), or after recognition of stalled ribosomes by messenger RNA surveillance factors such as Pelo, an eukaryotic Rf1 (eRf1) paralog. Notably, Abce1 is able to physically interact with eRf1 and directly influence its function in stop-codon recognition and peptidyl-transfer RNA hydrolysis. Failure to accomplish or fully complete this task, anticipated in a recessive mutant, would most probably negatively affect viral as well as host-plant protein synthesis. This in turn may result in slower infection progression, but may also negatively impact aspects of host plant development or its horticultural performances [33]. A screen-house study, carried out in this study, demonstrate that uninfected nearly-isogenic BC_4_F_3_ plants, homozygous for the *Pelo* allele derived from TY172, displayed a significant reduction in fruit size, and a small but not significant reduction in total fruit yield and harvest index compared to the recurrent susceptible parent (Table 5). This suggests that the presence of *ty-5* may exert a small penalty on yield. Whether these negative effects remain in more advanced BC generations is yet to be elucidated. Noteworthy, this discussion is limited to non-inoculated plants, to the determinate growth-habit characterizing TY172 and M-82 plants, and to a single genetic background (M-82), while excluding possible contribution that hybrid vigor may have towards increased yield in hybrid commercial plants carrying *ty-5*. It should be clarified, that under TYLCV-inoculation, *ty-5* has a clear advantage over susceptible plants [17, 23].

In a recent forward genetic screen for Drosophila mutants that are resistant to *Drosophila C virus* (DCV), a virus resistant line was found which had a deficient expression of the *Pelo* gene [34]. The Pelo deficiency was found to limit the high level synthesis of the DCV capsid protein. It was suggested that the reduction in synthesis of viral capsid is due to the role of Pelo in dissociation of stalled 80S ribosomes and clearance of aberrant viral RNA and proteins [34]. It should be noted however, that DCV is an RNA virus, completely different from TYLCV, which is a ssDNA virus.

To the best of our knowledge, *Pelo* and its protein product have never been implicated in virus resistance in plants and thus offer an alternative route to obtain such resistance. Our suggestion that its effect may be expressed through its role in protein translation machinery is promising. However, a direct interaction of Pelo with proteins involved in viral replication cannot be excluded.

## Materials and Methods

### Plant material and experimental layout

5,662 segregating F_2_, F_3_, F_4_, BC_1_F_1_, BC_1_F_2_, BC_1_F_3_, BC_1_F_4_, BC_1_F_5_, BC_2_F_2_, BC_2_F_3_ and BC_2_F_4_ seedlings originating from a cross between TY172 and M-82 (LA3475), were inoculated with TYLCV and genotyped with polymorphic markers spanning the *ty-5* locus. M-82 was chosen as the recurrent parent due to its determinate stature, facilitating working with large number of plants. The inoculation experiments were carried out in 8x16 sowing trays. Eight seedlings of the two parental lines were included in each tray while their F_1_ hybrid plants were included sporadically. Twenty-one days post inoculation (DPI), the seedlings were transplanted to the field or to 50-mesh screen-houses. Parental lines and their F_1_ hybrids were planted in a randomized block design, five or more plants in three blocks, while their segregating counterparts were planted at random. In the field plants were grown in rows, one m apart, allowing 50 cm space between plants, while in screen-houses in eight L pots, similarly arranged. Plants were grown using standard local cultural practices, including drip irrigation and fertilization.

The effect of the *Pelo* allele derived from TY172 on yield of non-inoculated segregating BC_4_F_3_ plants was estimated in a randomized-block screen-house experiment (three blocks, five plants per genotype per block). These BC_4_F_3_ plants, developed with M-82 as a recurrent parent and *Pelo* as the sole marker, were compared to M-82. Total fruit yield, fruit number, average fruit weight, plant weight and harvest index (fruit-yield/plant-weight ratio) were recorded for each plant.

Other lines and accessions used: 1. Heinz 1706—a TYLCV-susceptible determinate line obtained from the Tomato Genetics Resource Center (TGRC at http://tgrc.ucdavis.edu/). This line was initially used to sequence the tomato genome (SGN, http://solgenomics.net/); 2. R13—a TYLCV-susceptible indeterminate line (Hazera Genetics, Berurim, Israel); 3. LA1589, LA0441, PI 126926, PI 126930, and PI 390681—LA accessions were obtained from TGRC while PI accessions were obtained from the U.S. Department of Agriculture, Plant Genetic Resources Unit, Geneva, NY; 4. Seeds of the TYLCV-resistant hybrid Tyking were generously provided by Jaap Hoogstraten (Monsanto).

### Whitefly maintenance, plant inoculation and disease scoring

Whitefly colonies (*Bemisia tabaci*, biotype B), reared on cotton plants (*Gossypium hirsutum*), were used for TYLCV (Genbank accession No. X15656) inoculation as described [23, 35]. Thereafter, plants were sprayed with imidacloprid (Bayer, Leverkusen, Germany) and held in an insect-proof greenhouse at 26–32°C. TYLCV-induced symptoms were evaluated for each plant separately at 28 and 42 DPI according to a disease severity index (DSI) of 0-to-4 as follows: (0) no visible symptoms, inoculated plants show same growth and development as non-inoculated plants; (1) very slight yellowing of leaflet margins on apical leaf; (2) some yellowing and curling of leaflet ends; (3) a wide range of leaf yellowing, curling and cupping, with reduction in size, yet plants continue to develop and (4) very severe plant stunting and yellowing, pronounced cupping and curling, plants stop growth [21, 24].

### Genomic DNA extraction and polymorphism identification

Genomic DNA was extracted from individual plants according to [36]. A 4394 base-pair (bp) genomic region spanning the *Nac1* gene sequence in M-82 was blasted against the tomato sequence database at the Solanaceae Genomics Network (SGN, http://solgenomics.net/) and found fully homologous to the 8,734,372–8,738,765 bp region of the scaffold DNA sequence file SL2.40sc03604, and 2,854,539–2,858,932 bp region of the tomato chromosome 4 sequence in the DNA sequence file SL2.40ch04 (version SL2.40). Sequences in SL2.40sc03604 and SL2.40ch04 were used to design polymerase chain reaction (PCR) primers expected to amplify 800-to-900 bp fragments of the tomato genome spanning the *Nac1* gene region: first, approximately every 50 Kilo bp (Kbp), then every 10 Kbp and finally every 3 Kbp and at times less. Altogether, 257 such fragments were sequenced. Sequence analysis and locus-specific primer design were carried out with the DNAMAN sequence analysis software v4.1 (Lynnon BioSoft, Québec, Canada). DNA primers were purchased from Molecular Biology Center (Ness-Ziyyona, Israel).

The primers designed were used to PCR-amplify genomic DNA of TY172 and M-82 plants; amplification products were visualized by electrophoresis, extracted from the gel and directly sequenced according to [17]. These sequences were compared to the sequence of Heinz 1706 (http://solgenomics.net/) in order to detect single nucleotide polymorphisms (SNPs). These polymorphisms were further used to design polymorphic DNA markers, utilizing restriction endonucleases when necessary, that were analyzed for association with the resistance trait in segregating populations. Altogether, a core set of 27 polymorphic DNA markers, spanning the *ty-5* locus, were utilized by PCR (S1 Table). Following amplification, PCR products were digested and visualized by electrophoresis according to [17].

### Melting curve SNP genotyping for *Pelo*

We used a Melting curve SNP genotyping method (McSNP) with primers designated as McSNP F and R in S1 Table. Primers design was carried out by DYN R&D (Qesariyya, Israel). The McSNP genotyping reaction, described previously [37], was calibrated and carried out by DYN R&D using the LightCycler 480 instrument (Hoffmann-La Roche, Basel, Switzerland). Initial reaction conditions were: incubation at 95°C 10 min, followed by 50 cycles of 95°C 10 sec, a touch-down annealing 63°C→56°C 10 sec (-1.5°C per cycle) and 72°C 10 sec. Melting curve reaction conditions were: 95°C 1 min, 40°C 2 min, and 40–75°C degree, five acquisitions per degree. Results were analyzed using the LightCycler 480 SW 1.5 software (Hoffmann-La Roche), by DYN R&D utilizing its melt-curve analysis.

### Silencing of *Pelo*

Construction of *Pelo* silencing vector: to silence *Pelo*, a pHannibal vector [38] expressing a sense and anti-sense fragment of the gene was constructed in two steps. In the first step, a 576 bp fragment of the *Pelo* gene (cDNA coordinates 560 to 1136) was amplified by PCR using the forward primer S*Pelo*F-*Xho*I (5'-AGACTCGAGGACAATGTTCTACAGGCCTTTG-3'), containing a *Xho*I restriction site, and the reverse primer S*Pelo*R-*Kpn*I (5'-GACGGTACCCATCTCAAT GTCTTCCAGCTC-3'), containing a *Kpn*I site. This fragment was cloned into the unique *Xho*I and *Kpn*I sites present in the sense oriented arm of pHannibal. In the second step, the same 576 bp fragment of the gene was amplified by PCR performed with the forward primer S*Pelo*F-*Xba*I (5'-ATCTAGAGACAATGTTCTACAGGCCTTTG-3'), containing a *Xba*I site, and the reverse primer S*Pelo*R-*Cla*I (5'-CATCGATCATCTCAATGTC TTCCAGCTC-3'), containing a *Cla*I site. This fragment was cloned into the unique *Xba*I and *Cla*I sites present in the anti-sense oriented arm of pHannibal, thus creating pHannibal-*Pelo*.

To create a binary vector, pHannibal-*Pelo* was cloned under the cauliflower mosaic virus (35S) promoter and the nitric oxide synthase transcriptional terminator into the *Not*I site of the pBIN vector.

Transformations were carried out on cotyledon cuttings of TY172 and the susceptible R13 lines with *Agrobacterium tumefaciens* strain EHA105 as previously described [39]. R13 was chosen as the susceptible control due to its ease of transformation. Moreover, sequence analysis of *Pelo* in R13 revealed that it is identical to M-82.

### Over-expression of *Pelo*

*Pelo* cDNA was cloned from both TY172 and M-82 plants, and inserted into a pBIN vector under the control of the 35S promoter. To create a pBIN expression vector, the cassette containing the 35S promoter, omega enhancer, and the NOS terminator was cloned into the HindIII-EcoRI sites of pBINPLUS.

To create the plasmids pBIN*Pelo*-M-82 and pBIN*Pelo*-TY172, the *Pelo* gene was PCR amplified using the primers *Pelo*F (5’-CTAGGATCCatgaagattgttcgtagag-3’), containing a BamHI restriction endonuclease site, and *Pelo*R (5’-CTAGCGGCCGCATCACATCTCAATGTCTTC-3'), containing a NotI site. The amplification product was restricted with both BamHI and NotI and cloned into the appropriate sites of the pBIN vector. Transformations were carried out as described above.

### Characterization of transgenic plants

To validate incorporation of either the *Pelo* silencing construct or the over-expression constructs, DNA samples extracted from individual transformed plants served as templates in PCRs using a primer complementary to the 35S promoter (5’-CCTTCGCAAGACCCTTCCTCT-3’) and a primer complementary to 3’ of the *Pelo* gene sequence (5’-CTAGCGGCCGCATCACATCTCAATGTCTTC-3') for the silencing construct, while for the two *Pelo* transgenes a primer complementary to the *Pelo* gene sequence (5’-CTAGCGGCCGCATCACATCTCAATGTCTTC-3') was used. The PCRs were performed in a volume of 20 μl containing 15 ng of template DNA, 10 pmol of each primer, 0.2 mM of each dNTP, 2 mM MgCl_2_, 0.5 U of Taq DNA polymerase, and 1XPCR-buffer. The PCRs conditions were: 94°C 3 min, followed by 35 cycles of 94°C 30 s, 60°C 30 s, and 72°C 1 min. Final elongation was at 72°C 10 min. Amplification products were visualized by electrophoresis.

### Detection of TYLCV DNA copy number in plants

TYLCV DNA copy-number in plants was determined using quantitative Real-Time PCR (qRT-PCR). Total DNA was extracted from plant apices. TYLCV primers were designed using the PRIMER 3 procedure (http://workbench.sdsc.edu/): TYRT2F = 5'-GCTGATCTGCCATCGATTTG-3' and TYRT2R = 5'-GGTTCTTCGACCTGGTATC-3' forming a 147 bp amplicon. The qRT-PCR was carried out on a Corbett Rotor-Gene 6000 (Qiagen, Düesseldorf, Germany) with the following profile: 40 cycles of 95°C 10 s, 60°C 15 s, and 72°C 20 s; qRT-PCR reactions (12 μl volume) included 3 μl of plant DNA, 6 μl of SYBR Fast Universal Readymix Kit (Kapa Biosystems, Boston, MA), and 0.125 μM of each primer.

DNA extracted from non-infected plants and water served as negative controls. Each qRT-PCR reaction was run in duplicate, with 5 replications per treatment. DNA of each sample was extracted from three different infected plants. For standard curve, PCR amplicon was cloned into pGEM-T Easy (Promega, Madison, WI). The plasmids were extracted using Plasmid Miniprep kit (Qiagen) and linearized by digesting with *Pst*I. Gel-extracted fragments were quantified and used to create standard curves. Dilution series were performed by copy number following methods recommended by Applied Biosystems (Foster City, CA). Cycle threshold and copy number were determined using Corbett Rotor-Gene 6000 Series software. Amplification was followed by melt-curve analysis.

### *Pelo* relative transcript levels

*Pelo* relative transcript levels were determined by qRT-PCR. Total RNA was extracted from tomato apex using TRI-reagent (Sigma-Aldrich, St. Louis, MO) and DNA contaminants were digested with TURBO DNA-free DNAase (Ambion, Austin, TX). The remaining RNA was used as template for cDNA synthesis using the Revertaid first strand cDNA synthesis Kit (Fermentas).

*Pelo* primers were designed using the Primer 3 procedure. Primers used for the qRT-PCR reaction of the *Pelo* transcript level were: *Pelo*RTF = 5'-CCATGAGCGTCTGGCTATTC-3' and *Pelo*RTR = 5'-GGAGACATGCATTGACGAGA-3', forming a 150 bp amplicon.

The qRT-PCRs (12 μl volume) were performed as outlined above, with the following profile: 40 cycles of 95°C 10 s, 58°C 15 s, and 72°C 20 s. 18S ribosomal RNA was used as reference utilizing the following primers: 18SF = 5'-GCGACGCATCATTCAAATTTC-3' and 18SR = 5'-TCCGGAATCGAACCCTAATTC-3'.

qRT-PCR analyses were performed using the Rotor-Gene Q detection system and data was collected and analyzed with the Rotor-Gene 6000 software version 1.7.28 (Qiagen). Relative abundance of *Pelo* transcripts were calculated by the formula: 2^-(CT_Pelo-CT_18S)^, where CT represents the fractional cycle number at which the fluorescence crosses a fixed threshold (usually set on 0.1).

### Statistical analyses

The association between DNA markers and DSI scores were evaluated by analyses of variance and chi-square (χ^2^) with the JMP statistical discovery software (SAS Institute, Cary, NC). An excellent agreement was found between χ^2^ and the respective analyses of variance; therefore, analyses of variance are presented throughout this manuscript.

Differences in the relative abundance of the *Pelo* transcripts between genotypes were analyzed, following transformation to their natural logarithm, by analyses of variance using JMP software. Differences among means were statistically evaluated based on Tukey-Kramer Honestly Significant Difference test [40].

## Supporting Information

S1 Fig Genomic nucleotide sequence of the *Nac1* gene in the resistant TY172 line compared to the susceptible line M-82. Start (ATG) and stop (TAA) codons of the *Nac1* gene are highlighted with cyan and underlined; transcribed regions of the *Nac1* gene, including the 5' and 3' un-translated regions are highlighted with gray; nucleotide polymorphisms that differentiate between TY172 and M-82 are in red letters highlighted with yellow; the single-nucleotide polymorphism in the coding region of the *Nac1* gene that results in the Tyrosine^212^-to-Cysteine substitution of TY172 is highlighted with magenta; GenBank accession numbers for TY172 and M-82 are KC447282 and KC447283, respectively.
(PDF)

S2 Fig Amino-acid sequence of the *Nac1* gene in the resistant line TY172 compared to the susceptible line M-82. The Tyrosine^212^-to-Cysteine substitution of TY172 is highlighted with magenta; GenBank accession numbers for TY172 and M-82 are KC447279 and KC447280, respectively.
(PDF)

S3 Fig Analyses of association between DNA markers spanning the *ty-5* locus and TYLCV disease severity index (DSI) in 32 representative segregating populations originating from a single recombinant self-pollinated plant. In the ruler presented to the left of each analysis: yellow-shaded regions are homozygous for the alleles originated from the M-82 susceptible line (SS), red-shaded markers are homozygous (RR) for the alleles originated from the resistant line TY172 and gray-shaded regions are heterozygous (SR); the analysis of variance presented at the bottom of each population was carried out with different markers: the marker in red asterisks is the one that was used as an independent variable in each analysis; different superscript letters above means indicate statistically significant difference, *P*<0.05, between genotypes for each analysis separately. (A) shows the susceptible and resistant populations; (B) shows the segregating populations. Populations marked as susceptible are susceptible populations in which the marker used is not associated with DSI (no statistical difference was obtained between SS, SR and RR and thus the segregating regions do not contain the resistant gene); populations marked as resistant are resistant populations in which the marker used is also not associated with DSI (no statistical difference was obtained between SS, SR and RR and thus again the segregating regions do not contain the resistant gene); populations marked as associated segregating are populations in which the marker used is associated with DSI (statistical difference was obtained between RR and both SS and SR, thus the segregating regions do contain the resistant gene). DSI was determined at 28 and 42 DPI (days post inoculation), the DSI values presented is an average of both readings.
(PDF)

S4 Fig Genomic nucleotide sequence of the *Pelo* gene in the resistant TY172 line compared to the susceptible line M-82. Start (ATG) and stop (TAA) codons of *Pelo* are highlighted with cyan and underlined; transcribed regions of *Pelo*, including the 5' and 3' untranslated regions are highlighted with gray; nucleotide polymorphisms that differentiate between TY172 and both susceptible lines are in red letters highlighted with yellow; the single-nucleotide polymorphism in the coding region of *Pelo* that results in the substitution of Valine^16^ (susceptible lines) to a Glycine (resistant TY172 line) is highlighted with magenta; GenBank accession numbers for TY172 and M-82 are KC447287 and KC447288, respectively.
(PDF)

S5 Fig Silencing of *Pelo* in TYLCV-susceptible R13 plants. Transgenic and non-transgenic azygous control plants of lines TYT-413, 414 and 415 were inoculated with TYLCV and transplanted to a 50-mesh net-house; photographs were taken 28 days post inoculation. Note that the azygous control plants are showing TYLCV-induced disease symptoms of yellowing and cupping of leaves, especially in the plant apex, while the transgenic plants are not showing any disease symptoms (see arrows pointing to leaves showing disease symptoms).
(TIF)

S6 Fig Silencing of *Pelo* in TYLCV-resistant TY172 plants. Inoculated non-transgenic TY172 (A) and R13 (B) plants are compared to transgenic plants of lines TYT-411 (C) and TYT-412 (D); plants were inoculated with TYLCV and transplanted to a 50-mesh net-house; photographs were taken 28 days post inoculation. Note that the TY172 (A) and the transgenic plants (C and D) are not showing any disease symptoms while the R13 plant (B) is showing clear TYLCV-induced disease symptoms of yellowing and cupping of leaves, especially in the plant apex (see arrows pointing to leaves showing symptoms).
(TIF)

S1 Table DNA markers displaying polymorphisms between the resistant line TY172 and the susceptible counterpart M-82, on tomato chromosome 4. Markers are displayed according to their approximate distance in Kb from *Nac1*.
(PDF)
