# Supplementary material for: A Novel Route Controlling Begomovirus Resistance by the Messenger RNA Surveillance Factor Pelota
Source: PLoS Genet. 2015 Oct 8;11(10):e1005538. doi: 10.1371/journal.pgen.1005538 (PMC4598160; doi:10.1371/journal.pgen.1005538)
Supplement: S1 Fig — Start (ATG) and stop (TAA) codons of the Nac1 gene are highlighted with cyan and underlined; transcribed regions of the Nac1 gene, including the 5' and 3' un-translated regions are highlighted with gray; nucleotide polymorphisms that differentiate between TY172 and M-82 are in red letters highlighted with yellow; the single-nucleotide polymorphism in the coding region of the Nac1 gene that results in the Tyrosine212-to-Cysteine substitution of TY172 is highlighted with magenta; GenBank accession numbers for TY172 and M-82 are KC447282 and KC447283, respectively. (PDF) [file pgen.1005538.s001.pdf]

```
M-82            CTTTTTTGTCCTGACCTGACCGCCGTTTGAATTTTTTCTTATTGGATAATTATTATATTA 60
TY172           CTTTTTTGTCCTGACCTGACCGCCGTTTGAATTTTTTCTTATTGGATAATTATTATATTA 60
                ************************************************************

M-82            CTTTAACAGCCGACTTATTTAAAGTTTTTACTTTTTATGATATATCTTAAAATTCGCATA 120
TY172           CTTTAACAGCCGACTTATTTAAAGTTTTTACTTTTTATGATATATCTTAAAATTCGCATA 120
                ************************************************************

M-82            ATATTATTTATTGATATTCTCCTCACAAAAAAGATTTTTAGCGATAATTAATTAACAAAA 180
TY172           ATATTATTTATTGATATTCTCCTCACAAAAAAGATTTTTAGCGATAATTAATTAACAAAA 180
                ************************************************************

M-82            TAACTTAGTTGTCCATGAATATATTTTTTTAAATGCAAATTTATTAGTGAAAATTATATT 240
TY172           TAACTTAGTTGTCCATGAATATATTTTTTTAAATGCAAATTTATTAGTGAAAATTATATT 240
                ************************************************************

M-82            TTAGTATTTTTTATAAATATCACGAAAGTCTATAATAATAATTAGCTAATATTGATAAAG 300
TY172           TTAGTATTTTTTATAAATATCACGAAAGTCTATAATAATAATTAGCTAATATTGATAAAG 300
                ************************************************************

M-82            ATTTTAACCGTGAATTAAAAATATTTAGTAATTAAAATATATTTTTTCTTTTTTTCCCAA 360
TY172           ATTTTAACCGTGAATTAAAAATATTTAGTAATTAAAATATATTTTTTCTTTTTTTCCCAA 360
                ************************************************************

M-82            TCATATACTAAATATATCATAATATTAGTATGATTATAATAAATTATCATTAAGAAAGAA 420
TY172           TCATATACTAAATATATCATAATATTAGTATGATTATAATAAATTATCATTAAGAAAGAA 420
                ************************************************************

M-82            CAATATGAAAAGTATAAATTAGTTATTTTTTTAGTACCAATTAAAATAGGATTTTTTTTT 480
TY172           CAATATGAAAAGTATAAATTAGTTATTTTTTTAGTACCAATTAAAATAGGATTTTTTTTT 480
                ************************************************************

M-82            TAAAAAAAAATAAC-ATTAATTGGGACGGATATTATTATTTATTGGATCCTCAATGAGAA 539
TY172           TAAAAAAAAATAACCATTAATTGGGACGGATATTATTATTTATTGGATCCTCAATGAGAA 540
                ************** *********************************************

M-82            AGTGGTCCCCAGCAAGTGACGAGAGCAGTGACAAAATCATATGCACACGTGGGCGCATCT 599
TY172           AGTGGTCCCCAGCAAGTGACGAGAGCAGTGACAAAATCATATGCACACGTGGGCGCATCT 600
                ************************************************************

M-82            TTACAGGTGTTGTTCTCTCATTGGATGTTACTCTTCTATTGAGTCAATGGCTGCGGTCAA 659
TY172           TTACAGGTGTTGTTCTCTCATTGGATGTTACTCTTCTATTGAGTCAATGGCTGCGGTCAA 660
                ************************************************************

M-82            TCTAAAAGCAGTGAGGGTCCCACACACCCACTAGCTTAGACTCTTGATCACCAAGTAACT 719
TY172           TCTAAAAGCAGTGAGGGTCCCACACACCCACTAGCTTAGACTCTTGATCACCAAGTAACT 720
                ************************************************************

M-82            TTTTCTTAAAAGTACTCCCACCCCACCCCAACCCCCACACCCCCAACCCCTCACCCCACT 779
TY172           TTTTCTTAAAAGTACTCCCACCCCACCCCAACCCCCACACCCCCAACCCCTCACCCCACT 780
                ************************************************************

M-82            TAATGATTATGAAATAAAATAATTATTTCGTTCGTTTAAAAAATGATAAATTAGTTTGAC 839
TY172           TAATGATTATGAAATAAAATAATTATTTCGTTCGTTTAAAAAATGATAAATTAGTTTGAC 840
                ************************************************************

M-82            TTAGAACAGAGTTTACGAAAAGAAAAAATACTATTTAATCGTTATGTTAAATGTATCTAA 899
TY172           TTAGAACAGAGTTTACGAAAAGAAGAAATACTATTTAATCGTTATGTTAAATGTATCTAA 900
                ************************ ***********************************
```

```
M-82            ATGTCCTTTAATCTCGTGATCTTAAACATGTTATGTAGAAAGTTAAAATTAAAATATTGT 959
TY172           ATGTCCTTTAATCTCGTGATCTTAAACATGTTATGTAGAAAGTTAAAATTAAAATATTGT 960
                ************************************************************

M-82            CCAAAAAGAAAAGGAATCATTCTTTTTTAAACATACTAAAAAGGAAATATAATTTTTCTT 1019
TY172           CCAAAAAGAAAAGGAATCATTCTTTTTTAAACATACTATATAGGAAATATAATTTTTCTT 1020
                ************************************** * *******************

M-82            TTTGAAACGGAGAGAGTATATTTTTTTATAAAAAATTATGTGATCTCATTTGACTAGATA 1079
TY172           TTTGAAACGGAGAGAGTATATTTTTTTATAAAAAATTATGTGATCTCATTTGACTAGATA 1080
                ************************************************************

M-82            TAAATAATAATAATATATTGTAATTTTTCATGTTAGATTCTTAAATGAAGTAAAAATATT 1139
TY172           TAAATAATAATAATATATTGTAATTTTTCATGTTAGATTCTTAAATGAAGTAAAAATATT 1140
                ************************************************************

M-82            ATTAACCTTTCTGTATTAATATTCGATTTTTGAATCTTTATCGTAACAAGGGTAAAATTG 1199
TY172           ATTAACCTTTCTGTATTAATATTCGATTTTTGAATCTTTATCGTAACAAGGGTAAAATTG 1200
                ************************************************************

M-82            TGTCATCTTGACACATTTTTTTTTTATCTCTGTTCAAATTATTTCAGTCTATCTTTCCTT 1259
TY172           TGTCATCTTGACACATTTTTTTTTTATCTCTGTTCAAATTATTTCAGTCTATCTTTCCTT 1260
                ************************************************************

M-82            TTTTTATATATATTTTGTTACATAAGCTATTTCCAAGTTGTAAAACTTCGTTATCAAGCA 1319
TY172           TTTTTTTATATATTTTGTTACATAAGCTATTTCCAAGTTGTAAAACTTCGTTATCAAGCA 1320
                ***** ******************************************************

M-82            TATCTTTCATAGTATATTCACACATCTATTAGAATATACTCATCTCTACTATTTTTATCT 1379
TY172           TATCTTTCATAGTATATTCACACATCTATTAGAATATACTCATCTCTACTATTTTTATCT 1380
                ************************************************************

M-82            TTTAAACGTAGATATTCTTGAATATACTACATGAACAAAGTTGGTCTAGATTTAATTCAA 1439
TY172           TTTAAACGTAGATATTCTTGAATATACTACATGAACAAAGTTGGTCTAGATTTAATTCAA 1440
                ************************************************************

M-82            CAGTCACTCCATTTAAAAAAGAAGAAGATTTTACTTTTCTTTTTAATCGCCTCTAAAATG 1499
TY172           CAGTCACTCCATTTAAAAAAGAAGAAGATTTTACTTTTCTTTTTAATCGCCTCTAAAATG 1500
                ************************************************************

M-82            ACCAATTTTCTTTTTAGCAACATTTTCGTTTCGGCTTTTTACTTGACAACTGACATGTTT 1559
TY172           ACCAATTTTCTTTTTAGCAACATTTTCGTTTCGGCTTTTTACTTGACAACTGACATGTTT 1560
                ************************************************************

M-82            AACTCACAAGATTAACTATTAATTTGATATATTTGACACAATTTTAATTTATATTAATTC 1619
TY172           AACTCACAAGATTAACTATTAATTTGATATATTTGACACAATTTTAATTTATATTAATTC 1620
                ************************************************************

M-82            AAATTAGATCATTCACATAATTTTAAAAAAAAATATCTTACCAAAAAGCCTCAATACTTG 1679
TY172           AAATTAGATCATTCACATAATTTTAAAAAAAAATATCTTACCAAAAAGCCTCAATACTTG 1680
                ************************************************************

M-82            AATTTTATTGAATTATCAAACATATTATAGCAATACATATATATACAAAAATATATTATT 1739
TY172           AATTTTATTGAATTATCAAACATATTATAGCAATACATATATATACAAAAATATATTATT 1740
                ************************************************************

M-82            AAATACTTTATACTAATTTATTATTGTCGACATAATAACGGTGGTCCTGTTATTGCCTGG 1799
TY172           AAATACTTTATACTAATTTATTATTGTCGACATAATAACGGTGGTCCTGTTATTGCCTGG 1800
                ************************************************************
```

```
M-82            TTTCTGCTGTCAAACTCTACAGCACGTTTTATTTTATATTATTATACATACGACTAGTTT 1859
TY172           TTTCTGCTGTCAAACTCTACAGCACGTTTTATTTTATATTATTATACATACGACTAGTTT 1860
                ************************************************************

M-82            CAATCCACCACGTGCAAATTTACCCCCTATTTTTTCATCTATATATACACTTCAAACCCA 1919
TY172           CAATCCACCACGTGCAAATTTACCCCCTATTTTTTCATCTATATATACACTTCAAACCCA 1920
                ************************************************************

M-82            TCATTATTTTTTTGTCAAAGAAACTGAAACTAACACAAAGCAGGAGCAGGAGCAGCAACA 1979
TY172           TCATTATTTTTTTGTCAAAGAAACTGAAACTAACACAAAGCAGGAGCAGGAGCAGCAACA 1980
                ************************************************************

M-82            AACAGAGAGAAGAAAACAGAGGAAGATAAGAGGAAAATTTATCGAATTCGAATCGAGAGA 2039
TY172           AACAGAGAGAAGAAAACAGAGGAAGATAAGAGGAAAATTTATCGAATTCGAATCGAGAGA 2040
                ************************************************************

M-82            AAAGGGGAAGTGAAGTTGCGAAGAGTGAGAATTTCAAAGGAAATGAACAAAGGAGCAAAC 2099
TY172           AAAGGGGAAGTGAAGTTGCGAAGAGTGAGAATTTCAAAGGAAATGAACAAAGGAGCAAAC 2100
                ************************************************************

M-82            GGAAATCAGCAATTGGAGTTACCGGCGGGATTCAGATTCCATCCGACAGACGACGAATTG 2159
TY172           GGAAATCAGCAATTGGAGTTACCGGCGGGATTCAGATTCCATCCGACAGACGACGAATTG 2160
                ************************************************************

M-82            GTGCAGCACTATCTCTGCAGGAAATGCGCCGGACAGTCGATTGCTGTATCAATTATAGCT 2219
TY172           GTGCAGCACTATCTCTGCAGGAAATGCGCCGGACAGTCGATTGCTGTATCAATTATAGCT 2220
                ************************************************************

M-82            GAAATTGATCTTTACAAGTTTGATCCATGGCAGTTGCCTGGTAATTTTTGACTCTTCCTC 2279
TY172           GAAATTGATCTTTACAAGTTTGATCCATGGCAGTTGCCTGGTAATTTTTGACTCTTCCTC 2280
                ************************************************************


M-82            CAATTTTTCATAGCTTGCAAAGTCTAGGTCAAAAAAAAAATCGAAACAGATTAAGCTTCG 2339
TY172           CAATTTTTCATAGCTTGCAAAGTCTAGGTCAAAAAAAAAATCGAAACAGATTAAGCTTCG 2340
                ************************************************************

M-82            CGTTTGATCACAGTTTTTGGATCTGTTCCGCCATGGAATTTGAAAATCAGATCAAGTTCC 2499
TY172           CGTTTGATCACAGTTTTTGGATCTGTTCCGCCATGGAATTTGAAAATCAGATCAAGTTCC 2400
                ************************************************************

M-82            TTAAAACAAGTTTTTCGACTTATAGCATAGCAACACAACTTCAAATCTCAAAAATCACAA 2459
TY172           TTAAAACAAGTTTTTCGACTTATAGCATAGCAACACAACTTCAAATCTCATAAATCACAA 2460
                ************************************************** *********

M-82            TTTCAAAAACTCATAGATTTCAACTTCTATATTCAAACAGGAGCTAAATTTCGAATTTTA 2519
TY172           TTTCAAAAACTCATAGATTTCAACTTCTATATTCAAACAGGAGCTAAATTTCGAATTTTA 2520
                ************************************************************

M-82            AAAAATTATCTTCAAATATCCGTAAAAGTTAATCTTCGAACATTTTTAAGTTCCTAAAAC 2579
TY172           AAAAATTATCTTCAAATATCCGTAAAAGTTAATCTTCAAACATTTTTAAGTTCCTAAAAC 2580
                ************************************* **********************

M-82            TGTTTGGTTCTTTTTCTGAACTTTATTTTTTGTTGGTAATTGCAGAGAAGGCTTTGTACG 2639
TY172           TGTTTGGTTCTTTTTCTGAACTTTATTTTTTGTTGGTAATTGCAGAGAAGGCTTTGTACG 2640
                ************************************************************

M-82            GTGAAAAAGAGTGGTATTTTTTCTCACCAAGGGATAGAAAATATCCGAACGGTTCACGGC 2699
TY172           GTGAAAAAGAGTGGTATTTTTTCTCACCAAGGGATAGAAAATATCCGAACGGTTCACGGC 2700
                ************************************************************
```

```
M-82      CGAACCGAGCAGCAGGAACCGGTTATTGGAAGGCAACCGGAGCTGATAAACCGGTGGGAA 2759
TY172     CGAACCGAGCAGCAGGAACCGGTTATTGGAAGGCAACCGGAGCTGATAAACCGGTGGGAA 2760
          ************************************************************

M-82      AACCCAAAACCTTAGGGATAAAGAAGGCACTTGTGTTCTATGCCGGAAAAGCACCCAGAG 2819
TY172     AACCCAAAACCTTAGGGATAAAGAAGGCACTTGTGTTCTATGCCGGAAAAGCACCCAGAG 2820
          ************************************************************

M-82      GTATAAAAACAAATTGGATTATGCACGAGTACCGCCTCGCCAACGTGGACCGCTCTGCTG 2879
TY172     GTATAAAAACCAATTGGATTATGCACGAGTACCGCCTCGCCAACGTGGACCGCTCTGCTG 2880
          ********** *************************************************

M-82      GCAAGAACAATAACTTGAGGGTAAGTCCTTCTTCAGCTTTAATTTTTTATTTTCGGAGAT 2939
TY172     GCAAGAACAATAACTTGAGGGTAAGTCCTTCTTCAGCTTTAATTTTTTATTTTCGGAGAT 2940
          ************************************************************

M-82      GAGATTCAAGATTCGAGTAATATAGTATGGTAAAATTACTGAGTTTACGTGAACTATACT 2999
TY172     GAGATTCAAGATTCGAGTAATATAGTATGGTAAAATTACTGAGTTTACGTGAACTATACT 3000
          ************************************************************

M-82      CACATCTCTAATAGTGATATCTCCTAAGATTTACAATGTTTCAAATGAAAAATAGTGGAA 3059
TY172     CACATCTCTAATAGTGATATCTCCTAAGATTTACAATGTTTCAAATGAAAAATAGTGGAA 3060
          ************************************************************

M-82      GATTTCACTTTAATTATCAACAATTAGGTGTGGCACCCATTTAACATATAGTAATAAGTA 3119
TY172     GATTTCACTTTAATTATCAACAATTAGGTGTGGCACCCATTTAACATATAGTAATAAGTA 3120
          ************************************************************

M-82      AAATAAAATGGTCATTATATTCTGTAACTTATGAGTGCAAAATACAATTTGTAAATTGTT 3179
TY172     AAATAAAATGGTCATTATATTCTGTAACTTATGAGTGCAAAATACAATTTGTAAATTGTT 3180
          ************************************************************

M-82      TATTGATTATGTGATTTGACATTATTGCAGCTTGATGATTGGGTATTGTGTCGAATATAC 3239
TY172     TATTGATTATGTGATTTGACATTATTGCAGCTTGATGATTGGGTATTGTGTCGAATATAC 3240
          ************************************************************

M-82      AACAAGAAAGGCACACTTGAGAAGCATTACAATGTGGACAACAAGGAAACTACAAGCTTT 3299
TY172     AACAAGAAAGGCACACTTGAGAAGCATTACAATGTGGACAACAAGGAAACTACAAGCTTT 3300
          ************************************************************

M-82      GGAGAATTTGATGAAGAAATAAAACCAAAAATATTGCCCACACAATTAGCACCGATGCCA 3359
TY172     GGAGAATTTGATGAAGAAATAAAACCAAAAATATTGCCCACACAATTAGCACCGATGCCA 3360
          ************************************************************

M-82      CCACGGCCTCGATCGACACCAGCAAACGACTACTTTTATTTCGAGTCATCAGAGTCGATG 3419
TY172     CCACGGCCTCGATCGACACCAGCAAACGACTGCTTTTATTTCGAGTCATCAGAGTCGATG 3420
          ******************************* ****************************

M-82      ACTAGAATGCACACGACAAACTCGAGCTCTGGCTCAGAGCATGTCTTGTCGCCATGTGAC 3479
TY172     ACTAGAATGCACACGACAAACTCGAGCTCTGGCTCAGAGCATGTCTTGTCGCCATGTGAC 3480
          ************************************************************

M-82      AAGGAGGTTCAGAGCGCGCCCAAATGGGACGAAGACCACAGAAACACCCTTGATTTTCAG 3539
TY172     AAGGAGGTTCAGAGCGCGCCCAAATGGGACGAAGACCACAGAAACACCCTTGATTTTCAG 3540
          ************************************************************

M-82      CTAAACTATTTGGATGGTTTACTAAATGAACCATTTGAAACCCAAATGCAGCAGCAAATT 3599
TY172     CTAAACTATTTGGATGGTTTACTAAATGAACCATTTGAAACCCAAATGCAGCAGCAAATT 3600
          ************************************************************
```

```
M-82            TGCAACTTTGACCAGTTCAACAATTTCCAAGACATGTTCCTATACATGCAAAAACCTTAC 3559
TY172           TGCAACTTTGACCAGTTCAACAATTTCCAAGACATGTTCCTATACATGCAAAAACCTTAC 3660
                ************************************************************

M-82            TAAAATTGTATAAATTCATTGGATCTAAATTGAGTGTGATCCATGACATTTTCTTTGTTC 3719
TY172           TAAAATTGTATAAATTCATTGGATCTAAATTGAGTGTGATCCATGACATTTTCTTTGTTC 3720
                ************************************************************

M-82            TTTGGTGGTGTAGGTCAACTTTTTATTAAGTAGTTTAGAGAAGTACAAAATGCTAGTCAA 3779
TY172           TTTGGTGGTGTAGGTCAACTTTTTATTAAGTAGTTTAGAGAAGTACAAAATGCTAGTCAA 3780
                ************************************************************

M-82            ATTTGGTGGGCTACAGCACAAATGAGCCTTGATAAGCATAGCCAAAGAGTCGTATAGAAG 3839
TY172           ATTTGGTGGGCTACAGCACAAATGAGCCTTGATAAGCATAGCCAAAGAGTCATATAGAAG 3840
                *************************************************** ********

M-82            GGCTTATTATTATTGTAAGGTATGTAAAAACAAATGAAAATTTGTTAATATCAAGTTATC 3899
TY172           GGCTTATTATTATTGTAAGGTATGTAAAAACAAATGAAAATTTGTTAATATCAAGTTATC 3900
                ************************************************************

M-82            ATTCTTCAAATCTCTGTGATTATGACTCTCCTACACTGGTTCAAATAGAAATTAGCATAT 3959
TY172           ATTCTTCAAATCTCTGTGATTATGACTCTCCTACACTGGTTCAAATAGAAATTAGCATAT 3960
                ************************************************************

M-82            TTAAGAGCTCAGGGTTCAGGGGCAAAGTTATCGACATCGATGTGTCTGATTCATGTAGGC 4019
TY172           TTAAGAGCTCAGGGTTCAGGGGCAAAGTTATCGACATCGATGTGTCTGATTCATGTAGGC 4020
                ************************************************************

M-82            ATGAGAGCTAAAACTTTCTATTGTAAGATCCACAATAAGACAGTCGACTTGGGTGAAAAC 4079
TY172           ATGAGAGCTAAAACTTTCTATTGTAAGATCCACAATAAGACAGTCGACTTGGGTGAAAAC 4080
                ************************************************************

M-82            AATAGGAAAAAAGCCTAACAAATCATCAAGTTGAGTTGTTAACACTAAACAGTGACGAAG 4139
TY172           AATAGGAAAAAAGCCTAACAAATCATCAAGTTGAGTTGTTAACACTAAACAGTGACGAAG 4140
                ************************************************************

M-82            ACTCTTTCCTACATACAACATAGATGAATGTAACAAATAACAAGGCAAAACATCTTTAAC 4199
TY172           ACTCTTTCCTACATACAACATAGATGAATGTAACAAATAACAAGGCAAAACATCTTTAAC 4200
                ************************************************************

M-82            CTAGCAGTTAGTAAGATTTCGATTACCTCATATAAGAACTTCGCATCGTCCTTGATATAA 4259
TY172           CTAGCAGTTAGTAAGATTTCGATTACCTCATATAAGAACTTCGCATCGTCCTTGATATAA 4260
                ************************************************************

M-82            AATTTAAAAGAGCAAGAAACGAAATGAAATTCCTTACGTAAGGAGGAGTGTGGCTGCGCC 4319
TY172           AATTTAAAAGAGCAAGAAACGAAATGAAATTCCTTACGTAAGGAGGAGTGTGGCTGCGCC 4320
                ************************************************************

M-82            TCGTTTCATCATCATATGTTAGCAGACACTTATCCTTTCCAGCTTCCGCTACATACTAGT 4379
TY172           TCGTTTCATCATCATATGTTAGCAGACACTTATCCTTTCCAGCTTCCGCTACATACTAGT 4380
                ************************************************************

M-82            GAGTGCTGACTCCTC 4394
TY172           GAGTGCTGACTCCT- 4394
                **************
```
