# Supplementary material for: A Novel Route Controlling Begomovirus Resistance by the Messenger RNA Surveillance Factor Pelota
Source: PLoS Genet. 2015 Oct 8;11(10):e1005538. doi: 10.1371/journal.pgen.1005538 (PMC4598160; doi:10.1371/journal.pgen.1005538)
Supplement: S2 Fig — The Tyrosine212-to-Cysteine substitution of TY172 is highlighted with magenta; GenBank accession numbers for TY172 and M-82 are KC447279 and KC447280, respectively. (PDF) [file pgen.1005538.s002.pdf]

```
M-82            MNKGANGNQQLELPAGFRFHPTDDELVQHYLCRKCAGQSIAVSIIAEIDLYKFDPWQLPE 60
TY172           MNKGANGNQQLELPAGFRFHPTDDELVQHYLCRKCAGQSIAVSIIAEIDLYKFDPWQLPE 60
                ************************************************************

M-82            KALYGEKEWYFFSPRDRKYPNGSRPNRAAGTGYWKATGADKPVGKPKTLGIKKALVFYAG 120
TY172           KALYGEKEWYFFSPRDRKYPNGSRPNRAAGTGYWKATGADKPVGKPKTLGIKKALVFYAG 120
                ************************************************************

M-82            KAPRGIKTNWIMHEYRLANVDRSAGKNNNLRLDDWVLCRIYNKKGTLEKHYNVDNKETTS 180
TY172           KAPRGIKTNWIMHEYRLANVDRSAGKNNNLRLDDWVLCRIYNKKGTLEKHYNVDNKETTS 180
                ************************************************************

M-82            FGEFDEEIKPKILPTQLAPMPPRPRSTPANDYFYFESSESMTRMHTTNSSSGSEHVLSPC 240
TY172           FGEFDEEIKPKILPTQLAPMPPRPRSTPANDCFYFESSESMTRMHTTNSSSGSEHVLSPC 240
                ******************************* ****************************

M-82            DKEVQSAPKWDEDHRNTLDFQLNYLDGLLNEPFETQMQQQICNFDQFNNFQDMFLYMQKP 300
TY172           DKEVQSAPKWDEDHRNTLDFQLNYLDGLLNEPFETQMQQQICNFDQFNNFQDMFLYMQKP 300
                ************************************************************

M-82            Y 301
TY172           Y 301
```
