# Supplementary figures and images for: A Novel Route Controlling Begomovirus Resistance by the Messenger RNA Surveillance Factor Pelota
Source: PLoS Genet. 2015 Oct 8;11(10):e1005538. doi: 10.1371/journal.pgen.1005538 (PMC4598160; doi:10.1371/journal.pgen.1005538)

A

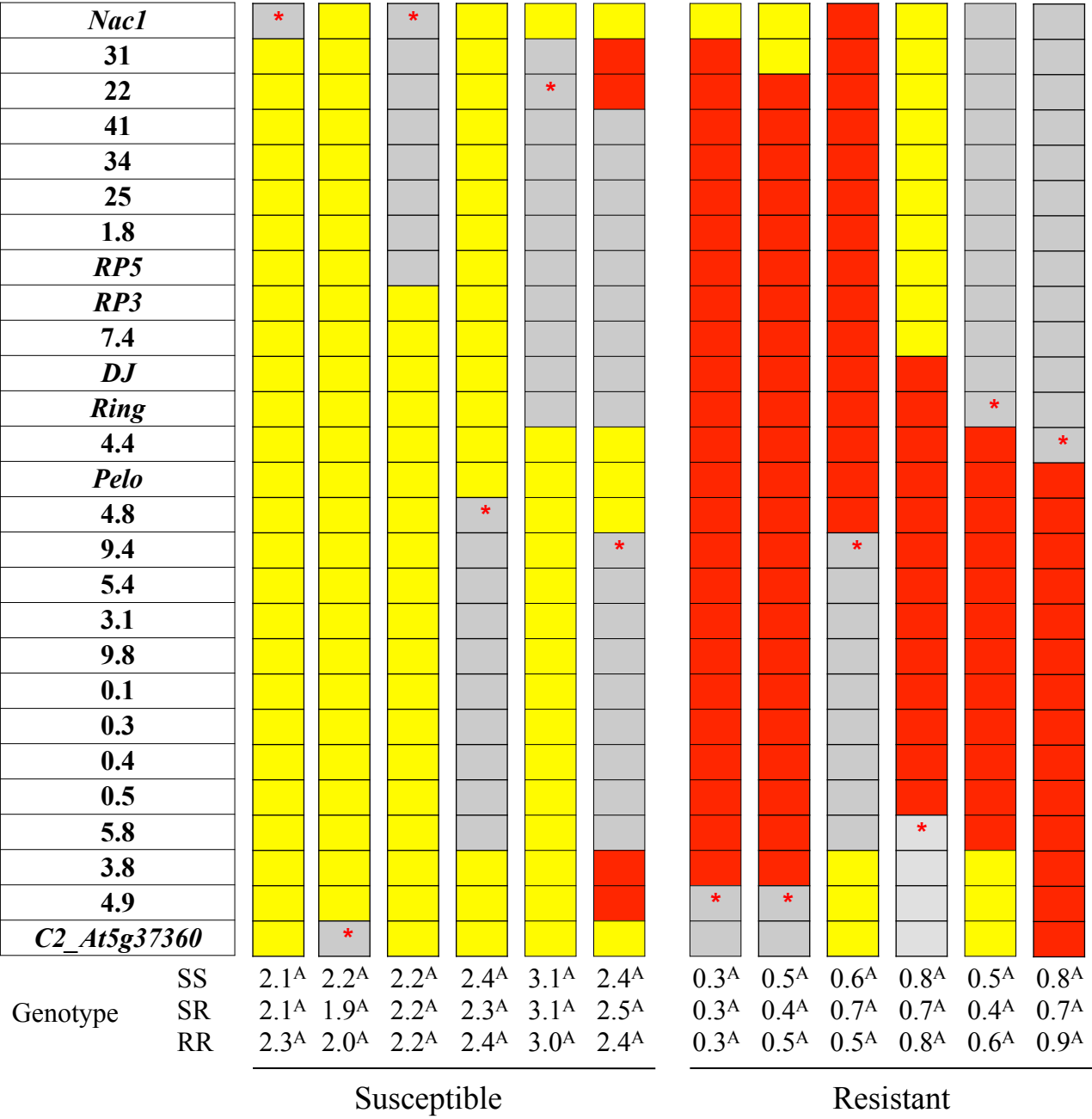

B

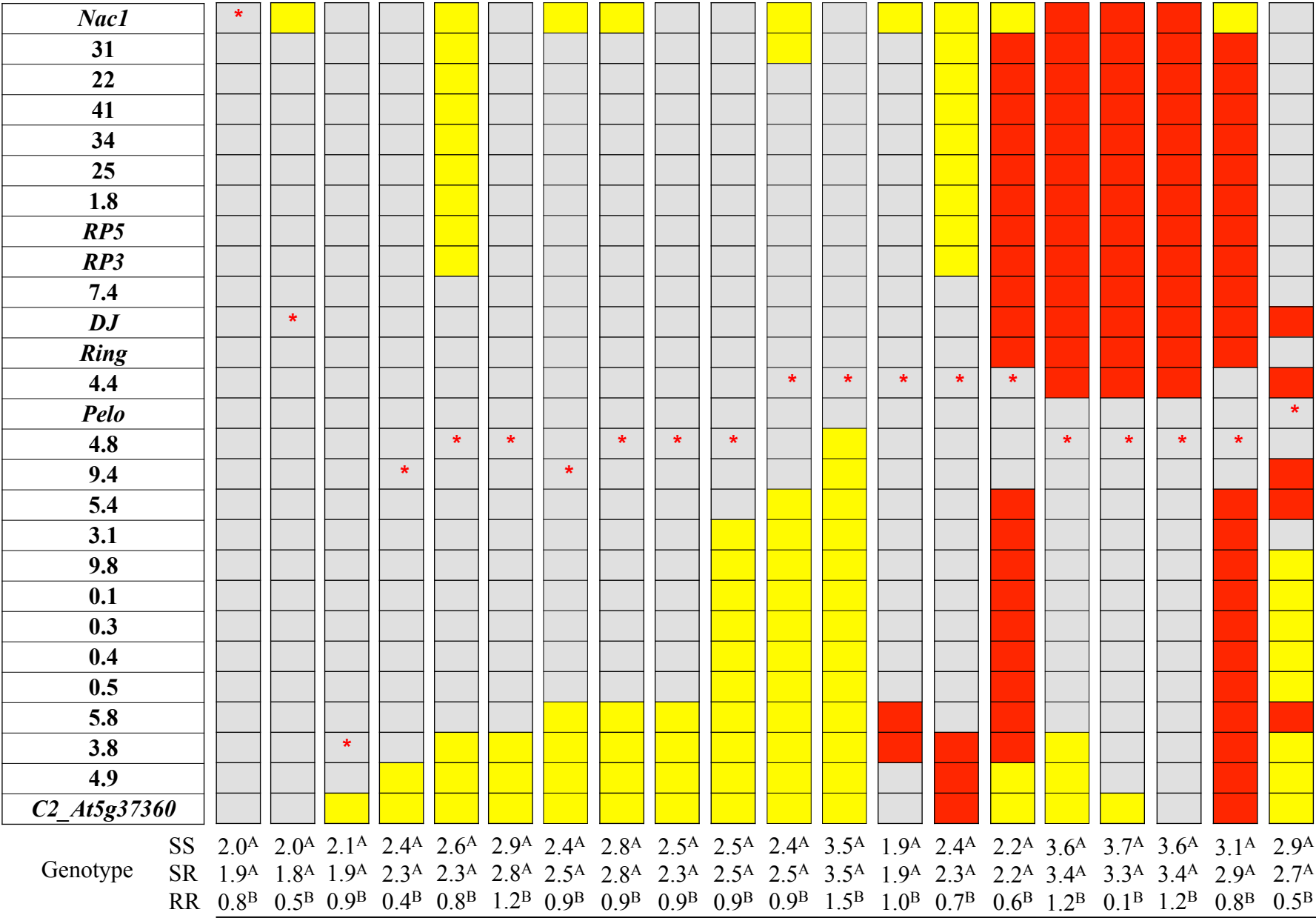

Supplement: S3 Fig — In the ruler presented to the left of each analysis: yellow-shaded regions are homozygous for the alleles originated from the M-82 susceptible line (SS), red-shaded markers are homozygous (RR) for the alleles originated from the resistant line TY172 and gray-shaded regions are heterozygous (SR); the analysis of variance presented at the bottom of each population was carried out with different markers: the marker in red asterisks is the one that was used as an independent variable in each analysis; different superscript letters above means indicate statistically significant difference, P<0.05, between genotypes for each analysis separately. (A) shows the susceptible and resistant populations; (B) shows the segregating populations. Populations marked as susceptible are susceptible populations in which the marker used is not associated with DSI (no statistical difference was obtained between SS, SR and RR and thus the segregating regions do not contain the resistant gene); populations marked as resistant are resistant populations in which the marker used is also not associated with DSI (no statistical difference was obtained between SS, SR and RR and thus again the segregating regions do not contain the resistant gene); populations marked as associated segregating are populations in which the marker used is associated with DSI (statistical difference was obtained between RR and both SS and SR, thus the segregating regions do contain the resistant gene). DSI was determined at 28 and 42 DPI (days post inoculation), the DSI values presented is an average of both readings. (PDF) [file pgen.1005538.s003.pdf]

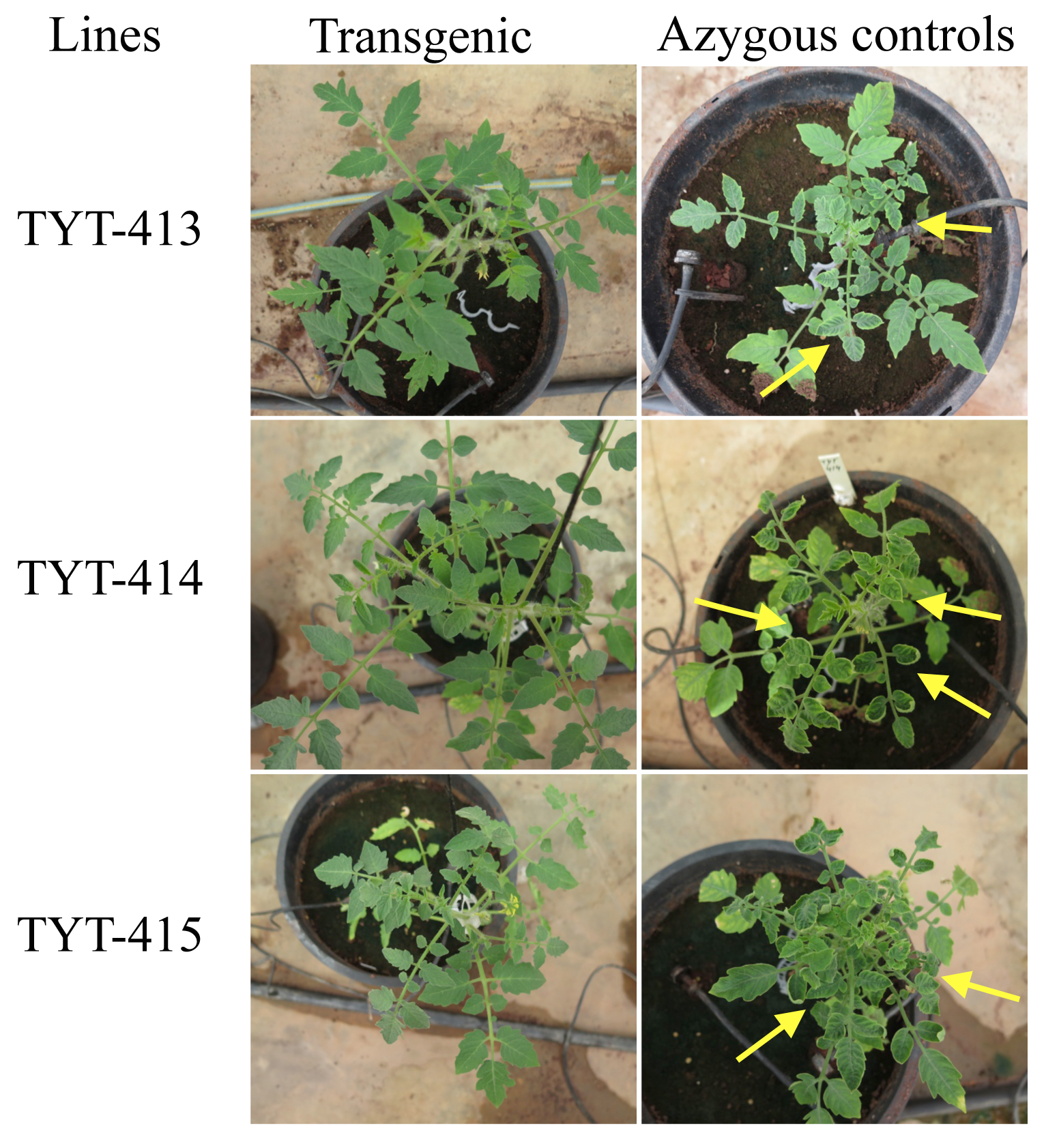

Supplement: S5 Fig — Transgenic and non-transgenic azygous control plants of lines TYT-413, 414 and 415 were inoculated with TYLCV and transplanted to a 50-mesh net-house; photographs were taken 28 days post inoculation. Note that the azygous control plants are showing TYLCV-induced disease symptoms of yellowing and cupping of leaves, especially in the plant apex, while the transgenic plants are not showing any disease symptoms (see arrows pointing to leaves showing disease symptoms). (TIF) [file pgen.1005538.s005.tif]

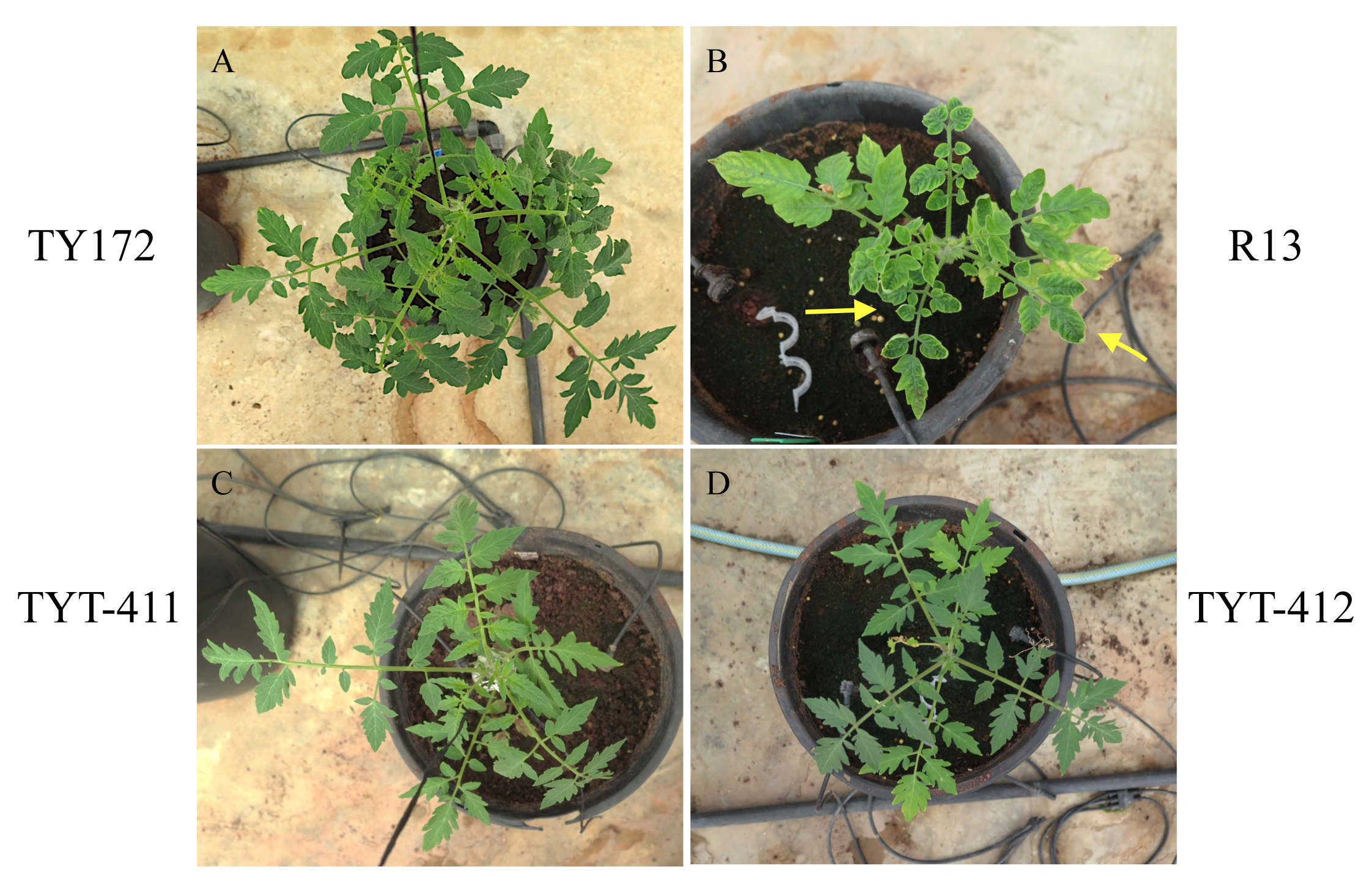

Supplement: S6 Fig — Inoculated non-transgenic TY172 (A) and R13 (B) plants are compared to transgenic plants of lines TYT-411 (C) and TYT-412 (D); plants were inoculated with TYLCV and transplanted to a 50-mesh net-house; photographs were taken 28 days post inoculation. Note that the TY172 (A) and the transgenic plants (C and D) are not showing any disease symptoms while the R13 plant (B) is showing clear TYLCV-induced disease symptoms of yellowing and cupping of leaves, especially in the plant apex (see arrows pointing to leaves showing symptoms). (TIF) [file pgen.1005538.s006.tif]
