# Supplementary material for: A Novel Route Controlling Begomovirus Resistance by the Messenger RNA Surveillance Factor Pelota
Source: PLoS Genet. 2015 Oct 8;11(10):e1005538. doi: 10.1371/journal.pgen.1005538 (PMC4598160; doi:10.1371/journal.pgen.1005538)
Supplement: S4 Fig — Start (ATG) and stop (TAA) codons of Pelo are highlighted with cyan and underlined; transcribed regions of Pelo, including the 5' and 3' untranslated regions are highlighted with gray; nucleotide polymorphisms that differentiate between TY172 and both susceptible lines are in red letters highlighted with yellow; the single-nucleotide polymorphism in the coding region of Pelo that results in the substitution of Valine16 (susceptible lines) to a Glycine (resistant TY172 line) is highlighted with magenta; GenBank accession numbers for TY172 and M-82 are KC447287 and KC447288, respectively. (PDF) [file pgen.1005538.s004.pdf]

```
M-82            GGACGTGTCATCTATAGTACTTAGTAGTAATATATCTTATATCTACATTACTTTTTTCAT 60
TY172           AGACGTGTCATCTATAGTACTTAGTAGTAATATATCTTATATCTACATTACTTTTTTCAT 60
                 ***********************************************************

M-82            GTCTGAAATTGACCTCTACAAATAGTTATAAATAACACTTTTGGCGTTTCAATTTTTTTA 120
TY172           GTCTGAAATTGACCTCTACAAATAGTTATAAATAACACTTTTGGCGTTTCAATTTTTTTA 120
                ************************************************************

M-82            ATAAATCAAAATTCTCAAATGTCATATTGGTAATTCTTATATTTATTTTAGCACTGCAAG 180
TY172           ATAAATCAAAATTCTCAAATGTCATATTGGTAATTCTTATATTTATTTTAGCACTGCAAG 180
                ************************************************************

M-82            TGTGTGTGTATATATATATATATATATATATATATTATTAGTAGGTTCGATGTTAAGAATCT 240
TY172           TGTGTGTGTATATATATATATATATATATATATATTATTAGTAGGTTCGATGTTAAGAATCT 240
                ************************************************************

M-82            AAAGGTCAAATTAGTCCAATTTATATTGTAGATTCATTTTTTCAAAGTAGTGCACTCATC 300
TY172           AAAGGTCAAATTAGTCCAATTTATATTGTAGATTCATTTTTTCAAAGTAGTGCACTCATC 300
                ************************************************************

M-82            CTTTCGAAATCCTAGATACTCTGTTTCAGATCCTATAATTGTAATAAAAAATTATGTTAA 360
TY172           CTTTCGAAATCCTAGATACTCTGTTTCAGATCCTATAATTGTAATAAAAAATTATGTTAA 360
                ************************************************************

M-82            TATGTATAAGTAGATAATAATGTATTTCGAACCTAATAAATTAAGAAGTATATGATTGAA 420
TY172           TATGTATAAGTAGATAATAATGTATTTCGAACCTAATAAATTAAGAAGTATATGATTGAA 420
                ************************************************************

M-82            AATACATAAATTTAACCTTTTGGATCCATCACTAAATTATACATCATTCTTGAGCATGAA 480
TY172           AATACATAAATTTAACCTTTTGGATCCATCACTAAATTATACATCATTCTTGAGCATGAA 480
                ************************************************************

M-82            AAAAAAAGACAACAAAACGAAATTCAAGTGAATATATGTGAACTGTGAAGTTATGGTTGG 540
TY172           AAAAAAAGACAACAAAACGAAATTCAAGTGAATATATGTGAACTGTGAAGTTATGGTTGG 540
                ************************************************************

M-82            CCCAATATTAGGCCCAATGTCATGGTTGGCTCAATTTTAGGCCCAATATCATGGTCCCCT 600
TY172           CCCAATATTAGGCCCAATGTCATGGTTGGCTCAATTTTAGGCCCAATATCATGGTCCCCT 600
                ************************************************************

M-82            TCAAATTGCTTCTCATGGGCCCAACATGGACCAAAAGAGGAAAAAAAAAACAAACTAGAG 660
TY172           TCAAATTGCTTCTCATGGGCCCAACATGGACCAAAAGAGGAAAAAAAAAACAAACTAGAG 660
                ************************************************************

M-82            GTTTATACTCGTACACAACGTAATCCCATAAGTAGAGTCTGAATAGAGTGATGTGTACAC 720
TY172           GTTTATACTCGTACACAACGTAATCCCATAAGTAGAGTCTGAATAGAGTGATGTGTACAC 720
                ************************************************************

M-82            AACTTTACCCTACCTTGTGAAGGTAGAGAGTTTGTTTCCGTTAAACCCTTTATTGGAGTA 780
TY172           AACTTTACCCTACCTTGTGAAGGTAGAGAGTTTGTTTCCGTTAAACCCTTTATTGGAGTA 780
                ************************************************************

M-82            AACATATCAAAAATCAAGTATATAAGGCAAAAACAGTTGAGAAAAAAAAAAACATGCTGAA 840
TY172           AACATATCAAAAATCAAGTATATAAGGCAAAAACAGTTGAGAAAAAAAAAAACATGCTGAA 840
                ************************************************************

M-82            AATAACTAAAAAGCCAACAAGTAGCAACACCAAATAATATGATAATCGAAAAGATATAAC 900
TY172           AATAACTAAAAAGCCAACAAGTAGCAACACCAAATAATATGATAATCGAAAAGATATAAC 900
                ************************************************************
```

```
M-82            CTATTGATAAGAGAACTAGACAATACTCAACTACATGCTAACTTTATACCCTAATTCTCA 960
TY172           CTATTGATAAGAGAACTAGACAATACTCAACTACATGCTAACTTTATACCCTAATTCTCA 960
                ************************************************************

M-82            ACCTTTATATCTTTGTATCTACAATCATGTTCTCGGTAAGCTGCAGATATTATATATTCA 1020
TY172           ACCTTTATATCTTTGTATCTACAATCATGTTCTCGGTAAGCTGCAGATATTATATATTCA 1020
                ************************************************************

M-82            GTCTAATTAGCTCACCCAAAAATCGTTTCAATCTACCTCTACCTCTTATGATACCTTACT 1080
TY172           GTCTAATTAGCTCACCCAAAAATCGTTTCAATCTACCTCTACCTCTTATGATACCTTACT 1080
                ************************************************************

M-82            ATATCCAACCTCTGACACTTCCTCGAAAGGGTATCCATACATTTTCTCTTCACATGTCCA 1140
TY172           ATATCCAACCTCTGACACTTCCTCGAAAGGGTATCCATACATTTTCTCTTCACATGTCCA 1140
                ************************************************************

M-82            AACCTTCCCAGCCTCATCTCTCTCATCCTGTCGACCACATAATTAAACCATTCTCACTTT 1200
TY172           AACCTTCCCAGCCTCATCTCTCTCATCCTGTCGACCACATAATTAAACCATTCTCACTTT 1200
                ************************************************************

M-82            ATCTCCAATAACTTCATTCTTGATCCTATTTCTTCTAAATGCACACACGTTTATTTTAAT 1260
TY172           ATCTCCAATAACTTCATTCTTGATCCTATTTCTTCTAAATGCACACACGTTTATTTTAAT 1260
                ************************************************************

M-82            ATCCTTATTTTTGCTACGTTTAATTTTTAAACATGTGATTTTTTTTTACTAATCAGTATT 1320
TY172           ATCCTTATTTTTGCTACGTTTAATTTTTAAACATGTGATTTTTTTTTACTAATCAGTATT 1320
                ************************************************************

M-82            AAGCTTTTAATGTTACATTTTAATCGCACAAAACTCTAGATGTAAATATATGCTTCATCC 1380
TY172           AAGCTTTTAATGTTACATTTTAATCGCACAAAACTCTAGATGTAAATATATGCTTCATCC 1380
                ************************************************************

M-82            ACCTGTTTCAATACTAAGATAACATCATCATCAGTCTCCTCCTAATTATTTTTTTTATAA 1440
TY172           ACCTGTTTCAATACTAAGATAACATCATCATCAGTCTCCTCCTAATTATTTTTTTTATAA 1440
                ************************************************************

M-82            CTAATTAAGTATGACATAAATTGATTTATTTTTCATTTGAATCAGGTCTAATCAAGTGGA 1500
TY172           CTAATTAAGTATGACATAAATTGATTTATTTTTCATTTGAATCAGGTCTAATCAAGTGGA 1500
                ************************************************************

M-82            AGAGGCAAAACAAAATTAGTTGAGACAATTTTTTTTTTAAAGTCATATTACATTAAAAAA 1560
TY172           AGAGGCAAAACAAAATTAGTTGAGACAATTTTTTTTTTAAAGTCATATTACATTAAAAAA 1560
                ************************************************************

M-82            AAAA-TAGGAAAAAATTACTTAAAGGGATACTAATAAATAACACTAATATTAGCAATACT 1619
TY172           AAAAATAGGAAAAAATTACTTAAAGGGATACTAATAAATAACACTAATATTAGCAATACT 1620
                **** *******************************************************

M-82            TCTCGTTTATTATCATTTATAGCCATATATAACAATACTATGATAAATGCAATACTATGT 1679
TY172           TCTCGTTTATTATCATTTATAGCCATATATAACAATACTATGATAAATGCAATACTATGT 1680
                ************************************************************

M-82            ATTAAAAAGTATATCATGTATGAAATATATGTGTATTATAACCGTTTTATAATATATTAT 1739
TY172           ATTAAAAAGTATATCATGTATGAAATATATGTGTATTATAACCGTTTTATAATATATTAT 1740
                ************************************************************

M-82            GTTTGTTTGGTAAGCTATTGACACATTGTATTATGTGTATTAATATGTTTTATCCACCAA 1799
TY172           GTTTGTTTGGTAAGCTATTGACACATTGTATTATGTGTATTAATATGTTTTATCCACCAA 1800
                ************************************************************
```

```
M-82      TAAAACTTGTATTATATGTATTTTATAAATTCTTCTTATTAAAACTGTATTATAAATGTA 1859
TY172     TAAAACTTGTATTATATGTATTTTATAAATTCTTCTTATTAAAACTGTATTATAAATGTA 1860
          ************************************************************

M-82      TTAAAAATTATCAAGTGAAAGTAAAATTATTACTATAAATGGTAAATACTTTTCTAATAT 1919
TY172     TTAAAAATTATCAAGTGAAAGTAAAATTATTACTATAAATGGTAAATACTTTTCTAATAT 1920
          ************************************************************

M-82      TACATATTTATGAAAGTTTTCCAAAAATATACTTTTTTTTTTAAAAAAAAAAATCCATAA 1979
TY172     TACATATTTATGAAAGTTTTCCAAAAATATACTTTTTTTTTAAAAAAAAAAAATCCATAA 1980
          ***************************************** ******************

M-82      ATATAAATTTGTTAAAAGAAAAGGTAAAGAAACATCATATATAACACAACAAATATATAA 2039
TY172     ATATAAATTTGTTAAAAGAAAAGGTAAAGAAACATCATATATAACACAACAAATATATAA 2040
          ************************************************************

M-82      AAAAATATTTTTAAAATAAAAATATACTAATTTTAAATTGCAAAATTGAGGGTATATTGG 2099
TY172     AAAAATATTTTTAAAATAAAAATATACTAATTTTAAATTGCAAAATTGAGGGTATATTGG 2100
          ************************************************************

M-82      TCATTTTCTCCCAAAAAAATAAAAATTCAAGTCCTTCTTCAACAAGGTTAGGAAAAACCT 2159
TY172     TCATTTTCTCCCAAAAAAATAAAAATTCAAGTCCTTCTTCAACAAGGTTAGGAAAAACCT 2160
          ************************************************************

M-82      AAAACATTAAAAGGACACAAACCGTTAAAACCTATAAAAAGTCTTTTCTCCATTGAACTG 2219
TY172     AAAACATTAAAAGGACACAAACCGTTAAAACCTATAAAAAGTCTTTTCTCCATTGAACTG 2220
          ************************************************************

M-82      AAGCAAATCTCATACGAAAAAAATTCTATAAAAAGTTGTTTAAATTCATCTTTTTTTCTG 2279
TY172     AAGCAAATCTCATACGAAAAAAATTCTATAAAAAGTTGTTTAAATTCATCTTTTTTTCTG 2280
          ************************************************************

M-82      CTATTTACATGCAAAATTATTGAATTTTTCATTAAGTTGATAAATTTGTTTCATTCAATA 2339
TY172     CTATTTACATGCAAAATTATTGAATTTTTCATTAAGTTGATAAATTTGTTTCATTCAATA 2340
          ************************************************************

M-82      TGAAGATTGTTCGTAGAGACTTTGTTCCTGATGGTTCTGGTAGTGTAAAGGTAACTTTTT 2399
TY172     TGAAGATTGTTCGTAGAGACTTTGTTCCTGATGGTTCTGGTAGTGGAAAGGTAACTTTTT 2400
          ********************************************* **************

M-82      TATCTCTATAATTGTTGTTTAAATCTATAATTCGAGTAATTTTCGTGATTTTTTGAAACC 2459
TY172     TATCTCTATAATTGTTGTTTAAATCTATAATTCGAGTAATTTTCGTGATTTTTTGAAACC 2460
          ************************************************************

M-82      CCAGATGAAGAAAACGTTAAAATTGATTTGGGTATTTTTTATTTTTTAATTTTCATGTGT 2519
TY172     CCAGATGAAGAAAACGTTAAAATTGATTTGGGTATTTTTTATTTTTTAATTTTCATGTGT 2520
          ************************************************************

M-82      TATACAGATAATTCCAGAAGAAGCTGATGATCTATGGGTTGCTTATAATCTGATAGCTGA 2579
TY172     TATACAGATAATTCCAGAAGAAGCTGATGATCTATGGGTTGCTTATAATCTGATAGCTGA 2580
          ************************************************************

M-82      AGGTGATACTGTATTAGCTGTTACTGTTAGGTATTGCACTTTTGCTCAATTTTATTAGTG 2639
TY172     AGGTGATACTGTATTAGCTGTTACTGTTAGGTATTGCACTTTTGCTCAATTTTATTAGTG 2640
          ************************************************************

M-82      TGAGGGCTTAGCATTATTAGCAATTTTTTTGGGATAAATAAGTAATTTTTATTCGCGTAT 2699
TY172     TGAGGGCTTAGCATTATTAGCAATTTTTTTGGGATAAATAAGTAATTTTTATTCGCGTAT 2700
          ************************************************************
```

```
M-82           GTAATAAGTTTGAGATTTTGTAGAAAAACATGTTTTTACTATAAGAAGCATTTAGTTTAT 2759
TY172          GTAATAAGTTTGAGATTTTGTAGAAAAACATGTTTTTACTATAAGAAGCATTTAGTTTAT 2760
               ************************************************************

M-82           ACTACTCCCTTACTCCGTTACAATTTGTTTGTTTGGTTTTGAATTGTCACGAGTTTTTTA 2819
TY172          ACTACTCCCTTACTCCGTTACAATTTGTTTGTTTGGTTTTGAATTGTCACGAGTTTTTTA 2820
               ************************************************************

M-82           AAAAAGAGAGTAAAGAACGACTTTTGAATCTCATGGTCTTTAAACTAAAGAGATTGTGGG 2879
TY172          AAAAAGAGAGTAAAGAACGACTTTTGAATCTCATGGTCTTTAAACTAAAGAGATTGTGGG 2880
               ************************************************************

M-82           ATGTACGGAATTTGGTCTTTTATCTTGTGCTATTAAATATGGTAGGTGGAAAGTCGAATA 2939
TY172          ATGTACGGAATTTGGTCTTTTATCTTGTGCTATTAAATATGGTAGGTGGAAAGTCGAATA 2940
               ************************************************************

M-82           GAAGAGTTGCCAAATAAGGAAAGAGACATTATTTTTGGAACAAACTAAAAAGAAAAGTAG 2999
TY172          GAAGAGTTGCCAAATAAGGAAAGAGACATTATTTTTGGAACAAACTAAAAAGAAAAGTAG 3000
               ************************************************************

M-82           GATAAACAAATTAAAACAGGGGGAGTATTTGGTTTCTCATCGGTGCTACAAGAATTACTA 3059
TY172          GATAAACAAATTAAAACAGGGGGAGTATTTGGTTTCTCATCGGTGCTACAAGAATTACTA 3060
               ************************************************************

M-82           AAAAGCTAGCGTCTTGCCTTTTTTAAAAAGAAATTTTGATCCTGAAAGATGGAGTTTTTA 3119
TY172          AAAAGCTAGCGTCTTGCCTTTTTTAAAAAGAAATTTTGATCCTGAAAGATGGAGTTTTTA 3120
               ************************************************************

M-82           ATTTGAATATGGATGTCACTTGCTAATTAGGTTCGAATTCTGTTCATTCGAAAGCTTGGA 3179
TY172          ATTTGAATATGGATGTCACTTGCTAATTAGGTTCGAATTCTGTTCATTCGAAAGCTTGGA 3180
               ************************************************************

M-82           GCTTTTTACTTTGTTTATAACTACATAGTTGATTCTCTACACTTGTTAATGTTTCTCATC 3239
TY172          GCTTTTTACTTTGTTTATAACTACATAGTTGATTCTCTACACTTGTTAATGTTTCTCATC 3240
               ************************************************************

M-82           CTGTTGAGTTGATTGAAAATTTATGTTTTTTGGATAACATATTAATTGCTCTCGTTCTCG 3299
TY172          CTGTTGAGTTGATTGAAAATTTATGTTTTTTGGATAACATATTAATTGCTCTCGTTCTCG 3300
               ************************************************************

M-82           TAAATGTCTAATTTCTGCATCAATTTTTGTTGTTTTATTCGTTGATTAGCTAATTCTTAG 3359
TY172          TAAATGTCTAATTTCTGCATCAATTTTTGTTGTTTTATTCGTTGATTAGCTAATTCTTAG 3360
               ************************************************************

M-82           AAAATGTAGTGTTCTGTGATGTGCGAAAGTAATCTATAAATAGTTAGACTCTAATCAGAT 3419
TY172          AAAATGTAGTGTTCTGTGATGTGCGAAAGTAATCTATAAATAGTTAGACTCTAATCAGAT 3420
               ************************************************************


M-82           TTGTGCTAAATTCTAGTTAAATTTGTCTAAATTGGCCTGAGATGAGTTTATATAAACTGT 3479
TY172          TTGTGCTAAATTCTAGTTAAATTTGTCTAAATTGGCCTGAGATGAGTTTATATAAACTGT 3480
               ************************************************************

M-82           GGAGTTACATGGTCAGTGAGGATTCATTTATACGACCTGAACTTGCTTGGACTGAGGTGT 3539
TY172          GGAGTTACATGGTCAGTGAGGATTCATTTATACGACCTGAACTTGCTTGGACTGAGGTGT 3540
               ************************************************************

M-82           TGTTGTTATTGTTGTTTGCTGAAATTGTGAATGACAATGTTACTATAAGGAAAATGGATT 3599
TY172          TGTTGTTATTGTTGTTTGCTGAAATTGTGAATGACAATGTTACTATAAGGAAAATGGATT 3600
               ************************************************************
```

```
M-82        TTGAGCATAGTGTGAGTCTCTACTGATTACACAGGTTGATAATAGTATTGTTATATTTGT 3659
TY172       TTGAGCATAGTGTGAGTCTCTACTGATTACACAGGTTGATAATAGTATTGTTATATTTGT 3660
            ************************************************************

M-82        TTTGCTTAAAGCTTAGGGGAAGACTCTTTTTTAATGTTGAGAGCAACTTTCTTTTGCAAA 3719
TY172       TTTGCTTAAAGCTTAGGGGAAGACTCTTTTTTAATGTTGAGAGCAACTTTCTTTTGCAAA 3720
            ************************************************************

M-82        TTATGCTATGTGGGTCTTTGCTGATTGTTAACACGGTTTAGCTTGTCACAACACAACCTA 3779
TY172       TTATGCTATGTGGGTCTTTGCTGATTGTTAACACGGTTTAGCTTGTCACAACACAACCTA 3780
            ************************************************************

M-82        CTGGAAGCTGGTTAAATTTTGCTTGTAATTTCTTGTAGGAAGGTCCTGAGGGAAGCTGCT 3839
TY172       CTGGAAGCTGGTTAAATTTTGCTTGTAATTTCTTGTAGGAAGGTCCTGAGGGAAGCTGCT 3840
            ************************************************************

M-82        TCTGGAGGAAGAGATGCTGAACGAGTGAAACTGAAATTGGAAATTAAAGTTGAGGTAAGG 3899
TY172       TCTGGAGGAAGAGATGCTGAACGAGTGAAACTGAAATTGGAAATTAAAGTTGAGGTAAGG 3900
            ************************************************************

M-82        ATATATTAGACATCCAGCATCATTCAGTTGTGGGGTGCGGGCCTTGGTTAGAACTTTGTT 3959
TY172       ATATATTAGACATCCAGCATCATTCAGTTGTGGGGTGCGGGCCTTGGTTAGAACTTTGTT 3960
            ************************************************************

M-82        TTAGCTTCCAGCATAAGGTGTTTTAATATAAATTGAAAGATAAAACTTTGAAATCAATTA 4019
TY172       TTAGCTTCCAGCATAAGGTGTTTTAATATAAATTGAAAGATAAAACTTTGAAATCAATTA 4020
            ************************************************************

M-82        ACTATAGAGAAGAATCTACTAAGGATAGAGAGGAGACTTTTCTTTGTCTTCCTTTTCCAA 4079
TY172       ACTATAGAGAAGAATCTACTAAGGATAGAGAGGAGACTTTTCTTTGTCTTCCTTTTCCAA 4080
            ************************************************************

M-82        TGTGGTCGAGATGAAGTATTTTTGGGCAGTTTGATGAATTTTGAGGATATCAATACAACC 4139
TY172       TGTGGTCGAGATGAAGTATTTTTGGGCAGTTTGATGAATTTTGAGGATATCAATACAACC 4140
            ************************************************************

M-82        CGTGTATGACATATGCATGATAAATTGTCACATAACAATGCTCCATTTCTAACTAATCTC 4199
TY172       CGTGTATGACATATGCATGATAAATTGTCACATAACAATGCTCCATTTCTAACTAATCTC 4200
            ************************************************************

M-82        AATAAATGCGATGTTGATAGTTTTGTTACCATAGTAATGATGACAATTGTAATGTGACTG 4259
TY172       AATAAATGCGATGTTGATAGTTTTGTTACCATAGTAATGATGACAATTGTAATGTGACTG 4260
            ************************************************************

M-82        GAAAGTAGGAAATAGATAGTGCTCTTTCTAGAGTTTTTATTTGAATATATTCCACTCCAA 4319
TY172       GAAAGTAGGAAATAGATAGTGCTCTTTCTAGAGTTTTTATTTGAATATATTCCACTCCAA 4320
            ************************************************************

M-82        TGTGGTATAATATAATCAATTTGGTGTTTTAGAATGTGGAGTATGACAAAGAAGGTTCTG 4379
TY172       TGTGGTATAATATAATCAATTTGGTGTTTTAGAATGTGGAGTATGACAAAGAAGGTTCTG 4380
            ************************************************************

M-82        CCTTGCGTATTCGCGGGAAGAATATTCTGGAGAATGAACATGTAAAGGTGTGTATTCTTC 4439
TY172       CCTTGCGTATTCGCGGGAAGAATATTCTGGAGAATGAACATGTAAAGGTGTGTATTCTTC 4440
            ************************************************************

M-82        AACTTAATCCTTTTGGATAAATTCCTAATATTGATGCTGCAACAAAAATGTATCTAATTA 4499
TY172       AACTTAATCCTTTTGGATAAATTCCTAATATTGATGCTGCAACAAAAATGTATCTAATTA 4500
            ************************************************************
```

```
M-82            TTTTATTGCAGATAGGGGCCTTTCACACTCTGGAAATTGAGCAACACAGACCTTTTGTGC 4559
TY172           TTTTATTGCAGATAGGGGCCTTTCACACTCTGGAAATTGAGCAACACAGACCTTTTGTGC 4560
                ************************************************************

M-82            TAAGAAAGGTACAATGCTGTGTTTTGATTCTTTTGCAACTATCACTCTGTTTTCTTTTAA 4619
TY172           TAAGAAAGGTACAATGCTGTGTTTTGATTCTTTTGCAACTATCACTCTGTTTTCTTTTAA 4620
                ************************************************************

M-82            AAATTTTGAGGTATATTATTTGCACTTTAAAAGCTATCAAGCTGGTGATATATGTTCTTT 4679
TY172           AAATTTTGAGGTATATTATTTGCACTTTAAAAGCTATCAAGCTGGTGATATATGTTCTTT 4680
                ************************************************************

M-82            AGGATTGCAGCTCTAACCTATGTTTCTCAGACTCTTCAAAAATGTCAACTGGTGCATGTC 4739
TY172           AGGATTGCAGCTCTAACCTATGTTTCTCAGACTCTTCAAAAATGTCAACTGGTGCATGTC 4740
                ************************************************************

M-82            GGATTCTCCAAAAATAGCGTGTTTTGGAATATCCGACATGGGTGCGGCATTGTAAGTGAA 4799
TY172           GGATTCTCCAAAAATAGCGTGTTTTGGAATATCCGACATGGGTGCGGCATTGTAAGTGAA 4800
                ************************************************************

M-82            GAGTCCGCAACTTAGGCTCTAACAAGTAAAAAAATCTGTAAAGTTATTCATGTAATTTAC 4859
TY172           GAGTCCGCAACTTAGGCTCTAACAAGTAAAAAAATCTGTAAAGTTATTCATGTAATTTAC 4860
                ************************************************************

M-82            ATTATTATTATAAAATCCCCGGAAAAAGAAGAAGAGTACCTTTTTTTTCTTTAATAACCT 4919
TY172           ATTATTATTATAAAATCCCCGGAAAAAGAAGAAGAGTACCTTTTTTTTCTTTAATAACCT 4920
                ************************************************************

M-82            TGGTATCCGGGCCAGTTTGTGCACACTTCGACCACTTCCACCAACACAGCTACCGCCTAC 4979
TY172           TGGTATCCGGGCCAGTTTGTGCACACTTCGACCACTTCCACCAACACAGCTACCGCCTAC 4980
                ************************************************************

M-82            AGGGTAACTCTTTCCATCAAGGTTTTGACAAATAAGAAGAAATTGTCTAGTGTTTTTCGC 5039
TY172           AGGGTAACTCTTTCCATCAAGGTTTTGACAAATAAGAAGAAATTGTCTAGTGTTTTTCGC 5040
                ************************************************************

M-82            CTCTGGTTGGATTTGAACCTAAGATCTTATGACTCTTAACTCACTTCATTGGCCGCTAGA 5099
TY172           CTCTGGTTGGATTTGAACCTAAGATCTTATGACTCTTAACTCACTTCATTGGCCGCTAGA 5100
                ************************************************************

M-82            CCATACCCCTTGGGTGCAGTTACTTGAAACTAAGTTTAATTTTTCCCGCTAAATTTCAGT 5159
TY172           CCATACCCCTTGGGTGCAGTTACTTGAAACTAAGTTTAATTTTTCCCGCTAAATTTCAGT 5160
                ************************************************************

M-82            TGTCCCGCCGCTGACACCCCTTCGAGGCATCTCGTGTTCCTGTCCTTTGACGTAGTTCAC 5219
TY172           TGTCCCGCCGCTGACACCCCTTCGAGGCATCTCGTGTTCCTGTCCTTTGACGTAGTTCAC 5220
                ************************************************************

M-82            ATCCTGCACGAAAGGTGTACATACGATACCTCCTTTCTGTCTCTAGATGGAACAACCCTT 5279
TY172           ATCCTGCACGAAAGGTGTACATACGATACCTCCTTTCTGTCTCTAGATGGAACAACCCTT 5280
                ************************************************************

M-82            CTACATTAATGATGATTCCACCTTGGTTATCATCATGACTAATTCCATGCCAAATAAGCC 5339
TY172           CTACATTAATGATGATTCCACCTTGGTTATCATCATGACTAATTCCATGCCAAATAAGCC 5340
                ************************************************************

M-82            AAGAAATAGCTACACATTTTCGGCACACACCAAAAAAGGGAACCCGAAGTATATTGTCGT 5399
TY172           AAGAAATAGCTACACATTTTCGGCACACACCAAAAAAGGGAACCCGAAGTATATTGTCGT 5400
                ************************************************************
```

```
M-82            TGATTTCTCATAAACAAGTGTACAATGGTTCGAAAATTATCCGAAAGCATTAATGCTTAT 5459
TY172           TGATTTCTCATAAACAAGTGTACAATGGTTCGAAAATTATCCGAAAGCATTAATGCTTAT 5460
                ************************************************************

M-82            TAAGCTATTATTAACTCCTATATTATACATTCTATGGACTTTGGTCTGAGGGGGCTAGTT 5519
TY172           TAAGCTATTATTAACTCCTATATTATACATTCTATGGACTTTGGTCTGAGGGGGCTAGTT 5520
                ************************************************************

M-82            TCTTTTCTCAGCTTGTTTCTTTGTAGTTTGAAGGATCTATGCCCCCTAGTTCGTCCAACA 5579
TY172           TCTTTTCTCAGCTTGTTTCTTTGTAGTTTGAAGGATCTATGCCCCCTAGTTCGTCCAACA 5580
                ************************************************************

M-82            CAGGTAGGGCTGAGGCATGATAAGATAAGTGACGGTTTGTGTAGCCTAGGTAATATATAC 5639
TY172           CAGGTAGGGCTGAGGCATGATAAGATAAGTGACGGTTTGTGTAGCCTAGGTAATATATAC 5640
                ************************************************************

M-82            TTAAGTGATGCAACAATATAAGTTCAGGTAGGGGAAATTCTTCTCGTACACATAGGAGTA 5699
TY172           TTAAGTGATGCAACAATATAAGTTCAGGTAGGGGAAATTCTTCTCGTACACATAGGAGTA 5700
                ************************************************************


M-82            CATGGAATTTGGTAAGATTTCTTGATTTTTTTCTGCATTATACAAAGTTGCTGCATAGAT 5759
TY172           CATGGAATTTGGTAAGATTTCTTGATTTTTTTCTGCATTATACAAAGTTGCTGCATAGAT 5760
                ************************************************************

M-82            TAAGTATAACTAAATAAGTGTCTTTTTATAATTAGAGGCTAATGAGTCATAGAAGTTGAA 5819
TY172           TAAGTATAACTAAATAAGTGTCTTTTTATAATTAGAGGCTAATGAGTCATAGAAGTTGAA 5820
                ************************************************************

M-82            TTAGGAGATAATCATTTGTAGTTGTAACTAGAGGTTTCTATAGGAAATTGAGGAGTATCC 5879
TY172           TTAGGAGATAATCATTTGTAGTTGTAACTAGAGGTTTCTATAGGAAATTGAGGAGTATCC 5880
                ************************************************************

M-82            CAATCGAAATATAAATGCCTAAAAATGTCTGGGCTAAGAATTACATGTGAACTTGATGTC 5939
TY172           CAATCGAAATATAAATGCCTAAAAATGTCTGGGCTAAGAATTACATGTGAACTTGATGTC 5940
                ************************************************************

M-82            TGGGCTAAGAATTACATGTGAACTTGAAATTATATTGATCGGGTACAGGTCGAATGAAAT 5999
TY172           TGGGCTAAGAATTACATGTGAACTTGAAATTATATTGATCGGGTACAGGTCGAATGAAAT 6000
                ************************************************************

M-82            TTTCCATAATTTCTTTTACTACTGGATTTATTACTTAAAAGGGTATAAACTTATCAAAAT 6059
TY172           TTTCCATAATTTCTTTTACTACTGGATTTATTACTTAAAAGGGTATAAACTTATCAAAAT 6060
                ************************************************************

M-82            AAGATTTCTTAGAAGGTATGCGTGCTTTTATTTAAAACTTTTGTTTATGTATAATCCTAT 6119
TY172           AAGATTTCTTAGAAGGTATGCGTGCTTTTATTTAAAACTTTTGTTTATGTATAATCCTAT 6120
                ************************************************************

M-82            CTAATGCATGTACATGAATTATTTTGAATTTATTTTTACTCAAAGTAATCGTTATTTTCC 6179
TY172           CTAATGCATGTACATGAATTATTTTGAATTTATTTTTACTCAAAGTAATCGTTATTTTCC 6180
                ************************************************************

M-82            TGTGTCATCTTCCTTATTATTTTTGTATATTCTATTTGTTTAGCAAATATATGAGCATCT 6239
TY172           TGTGTCATCTTCCTTATTATTTTTGTATATTCTATTTGTTTAGCAAATATATGAGCATCT 6240
                ************************************************************

M-82            TGTTTGTTTCAATCTTAGTCTTACCTGAACATAGTTGATTTATACGAAAGCTTATACATA 6299
TY172           TGTTTGTTTCAATCTTAGTCTTACCTGAACATAGTTGATTTATACGAAAGCTTATACATA 6300
                ************************************************************
```

```
M-82        TATGAGTTCTGACTTGCATTATCTGATTTAGGTGGTCTGGGACTCACTGGCACGGGAGGT 6359
TY172       TATGAGTTCTGACTTGCATTATCTGATTTAGGTGGTCTGGGACTCACTGGCACGGGAGGT 6360
            ************************************************************

M-82        TCTTCGTCAAGCTTCTGGTATTTTTCACAGTGCACTTTAACAAAGTTATATTTTTATTTG 6419
TY172       TCTTCGTCAAGCTTCTGGTATTTTTCACAGTGCACTTTAACAAAGTTATATTTTTATTTG 6420
            ************************************************************

M-82        TTCAAGTGTTGCCATTAACTTTATCTGCAATATTAGGCATTTTAGTTCATAGCCTGACTT 6479
TY172       TTCAAGTGTTGCCATTAACTTTATCTGCAATATTAGGCATTTTAGTTCATAGCCTGACTT 6480
            ************************************************************

M-82        TTCATCCATAACTTATCAAGTTCTATAACATGTTGCTGCTTCTTTTTAGTACATTGACAT 6539
TY172       TTCATCCATAACTTATCAAGTTCTATAACATGTTGCTGCTTCTTTTTAGTACATTGACAT 6540
            ************************************************************

M-82        GATTGTGAAACCACTTTATAGGTGCATTATTCTTAGATGGATTGTTTCAGTCTAGGTGTC 6599
TY172       GATTGTGAAACCACTTTATAGGTGCATTATTCTTAGATGGATTGTTTCAGTCTAGGTGTC 6600
            ************************************************************

M-82        TTTTAGCCCTTATGTTGCAGAGGTTTTCTTCTTTACTCGTACACTTGCAGTGAGAACATG 6659
TY172       TTTTAGCCCTTATGTTGCAGAGGTTTTCTTCTTTACTCGTACACTTGCAGTGAGAACATG 6660
            ************************************************************

M-82        ATTATTCGCTGCAAGTACATGTGTGTTGCAGAGTGCTTTTGAACTGATGAGAAGTTTCCT 6719
TY172       ATTATTCGCTGCAAGTACATGTGTGTTGCAGAGTGCTTTTGAACTGATGAGAAGTTTCCT 6720
            ************************************************************

M-82        ATATAATTGTTGATATTCAGCTATATTGATTCCAACATGGATATGTGTTTGTAACTTTGT 6779
TY172       ATATAATTGTTGATATTCAGCTATATTGATTCCAACATGGATATGTGTTTGTAACTTTGT 6780
            ************************************************************

M-82        ACTGCTCTTAGATTTACCGTTTCAATATGCTGAATACGGTTTTCCTTTTTGATGATTCAG 6839
TY172       ACTGCTCTTAGATTTACCGTTTCAATATGCTGAATACGGTTTTCCTTTTTGATGATTCAG 6840
            ************************************************************

M-82        ATCCATCTGCAAGTGCTGATCTGGCTGTGGTTCTGATGCAAGAAGGATTGGCACACATTC 6899
TY172       ATCCATCTGCAAGTGCTGATCTGGCTGTGGTTCTGATGCAAGAAGGATTGGCACACATTC 6900
            ************************************************************

M-82        TTCTTATTGGTAAAAGGTAAGCTTGACAATCTCATAGTCCTATGAATACAAGTTTTAAAC 6959
TY172       TTCTTATTGGTAAAAGGTAAGCTTGACAATCTCATAGTCCTATGAATACAAGTTTTAAAC 6960
            ************************************************************

M-82        AGATCTTGAGCATCTCCTTTTCTTGTATTTAAACGAGATGTAATAGTTTAAAAGTCGAAT 7019
TY172       AGATCTTGAGCATCTCCTTTTCTTGTATTTAAACGAGATGTAATAGTTTAAAAGTCGAAT 7020
            ************************************************************

M-82        GATTATGTCAGAATTTCTATATGTTGAGGCTGAGTTAAGTGTTAACTAGATTGTATGGCA 7079
TY172       GATTATGTCAGAATTTCTATATGTTGAGGCTGAGTTAAGTGTTAACTAGATTGTATGGCA 7080
            ************************************************************

M-82        TAATTTTACCTTATGTACTTGTTACTGTTGTCTGAACAGTGTGACTATCACCCGTTCTCG 7139
TY172       TAATTTTACCTTATGTACTTGTTACTGTTGTCTGAACAGTGTGACTATCACCCGTTCTCG 7140
            ************************************************************

M-82        TATAGAGTCTTCTATACCGCGCAAGCATGGACCGGCTATTGCAGGTTATGATAAGGTGAG 7199
TY172       TATAGAGTCTTCTATACCGCGCAAGCATGGACCGGCTATTGCAGGTTATGATAAGGTGAG 7200
            ************************************************************
```

```
M-82      TCTCTTACTTCTTTTTGTTTCATCTTTTGTATAATTTAATTATTTTGAACATGACGTCAA 7259
TY172     TCTCTTACTTCTTTTTGTTTCATCTTTTGTATAATTTAATTATTTTGAACATGACGTCAA 7260
          ************************************************************

M-82      GTGAAATTGTGTTCTTAATTTTGATTTGCAGGCGTTAAATAAATTCTTTGACAATGTTCT 7319
TY172     GTGAAATTGTGTTCTTAATTTTGATTTGCAGGCGTTAAATAAATTCTTTGACAATGTTCT 7320
          ************************************************************

M-82      ACAGGTAGACTTTTGTCAACTTTCTTGATGTTGCTTAATTTCCAGAAGCAATATGTTATA 7379
TY172     ACAGGTAGACTTTTGTCAACTTTCTTGATGTTGCTTAATTTCCAGAAGCAATATGTTATA 7380
          ************************************************************

M-82      GGTTCTTATTTCTGTTGCAGGCCTTTGTCAAGCATGTTGATTTCAAAGTAGTTCGCTGTG 7439
TY172     GGTTCTTATTTCTGTTGCAGGCCTTTGTCAAGCATGTTGATTTCAAAGTAGTTCGCTGTG 7440
          ************************************************************

M-82      CTGTGATTGCAAGTCCAGGATTCACCAAGGTATTTTTTGTATAGTTACACTTCTTAGCTA 7499
TY172     CTGTGATTGCAAGTCCAGGATTCACCAAGGTATTTTTTGTATAGTTACACTTCTTAGCTA 7500
          ************************************************************

M-82      GTCATACTTTTATGCTATGTTACAAGGGGTAGAACTTGCATATCTAATTATATCTGTACT 7559
TY172     GTCATACTTTTATGCTATGTTACAAGGGGTAGAACTTGCATATCTAATTATATCTGTACT 7560
          ************************************************************

M-82      ATGCATATGTTAATTCAGTTGTGACATTTAAACTTTTTTGTGTTGAATGTGCAGGATCAG 7619
TY172     ATGCATATGTTAATTCAGTTGTGACATTTAAACTTTTTTGTGTTGAATGTGCAGGATCAG 7620
          ************************************************************

M-82      TTTCATCGTCACCTGTTGTTGGAAGCCGAGAGGAAGCAACTAAGACCTATAATAGAAAAT 7679
TY172     TTTCATCGTCACCTGTTGTTGGAAGCCGAGAGGAAGCAACTAAGACCTATAATAGAAAAT 7680
          ************************************************************

M-82      AAGTCACGCATAATTCTTGTCCATACAACCTCGGGATACAAGTATGCCCTTCTTTCTCTC 7739
TY172     AAGTCACGCATAATTCTTGTCCATACAACCTCGGGATACAAGTATGCCCTTCTTTCTCTC 7740
          ************************************************************

M-82      TCTCCTTGCCCTTCATCTGACATCTCAAAACGAGTCAATACATTTTTGTGCAAACCATAT 7799
TY172     TCTCCTTGCCCTTCATCTGACATCTCAAAACGAGTCAATACATTTTTGTGCAAACCATAT 7800
          ************************************************************

M-82      GATGTTAGACATGCGTGCTAGTCTAAAATTGACTAATATGTAGCAATACATTTTTGTGTA 7859
TY172     GATGTTAGACATGCGTGCTAGTCTAAAATTGACTAATATGTAGCAATACATTTTTGTGTA 7860
          ************************************************************

M-82      CGCGATTCTCTCGTCAGTACTGCTATTTTAGTACAAATCGCCTTTATTATTTGTCTATTG 7919
TY172     CGCGATTCTCTCGTCAGTACTGCTATTTTAGTACAAATCGCCTTTATTATTTGTCTATTG 7920
          ************************************************************

M-82      TAGTTGATATGTAAAAGTTCAATTATTATCCAGCACGGGTGTTTAAGGCCGAGAGTTTTT 7979
TY172     TAGTTGATATGTAAAAGTTCAATTATTATCCAGCACGGGTGTTTAAGGCCGAGAGTTTTT 7980
          ************************************************************

M-82      TTTTTCCCTCGTTTTACAGACATAGTTTGAAAGAGGTTATGGATGCCCCAAATGTAATGA 8039
TY172     TTTTTCCCTCGTTTTACAGACATAGTTTGAAAGAGGTTATGGATGCCCCAAATGTAATGA 8040
          ************************************************************

M-82      CTATGATAAAAGATACAAAAGCTGCCAAAGAGGTACCTTCTGACCCTTGTCCAACTTGAT 8099
TY172     CTATGATAAAAGATACAAAAGCTGCCAAAGAGGTACCTTCTGACCCTTGTCCAACTTGAT 8100
          ************************************************************
```

```
M-82      ATGATCTTTAATCTTTATTCTTGTGTTTGTTCAAGTCTTTTTTCGTATTTTTTGGGAATA 8159
TY172     ATGATCTTTAATCTTTATTCTTGTGTTTGTTCAAGTCTTTTTTCGTATTTTTTGGGAATA 8160
          ************************************************************

M-82      ACTGTTTTCCTTTCTCTTCCAATCTTAATCAGGTTCAAGCCCTAAAGGATTTTTTCAACA 8219
TY172     ACTGTTTTCCTTTCTCTTCCAATCTTAATCAGGTTCAAGCCCTAAAGGATTTTTTCAACA 8220
          ************************************************************

M-82      TGCTTTCAAATGTTAGGTTCTATCCTTGGTCTCAATGTTCTATTTATATTTTTCTTAATA 8279
TY172     TGCTTTCAAATGTTAGGTTCTATCCTTGGTCTCAATGTTCTATTTATATTTTTCTTAATA 8280
          ************************************************************

M-82      ACTTTGGATGTTTCAGATTTTTATCTATCATATGCTAAGAGTAAACACAGATTGTTTGGT 8339
TY172     ACTTTGGATGTTTCAGATTTTTATCTATCATATGCTAAGAGTAAACACAGATTGTTTGGT 8340
          ************************************************************

M-82      TTTGAATTTATTTGTTTGCTTTTTTTGGTGTTTATCGGTTATCTTGGGTTCTGTAAGAGG 8399
TY172     TTTGAATTTATTTGTTTGCTTTTTTTGGTGTTTATCGGTTATCTTGGGTTCTGTAAGAGG 8400
          ************************************************************

M-82      CCTGAGAGTTTCAAATAAAAGTTTGAGGTGTTTAACTATGCATGACGGATATCCCTGTTT 8459
TY172     CCTGAGAGTTTCAAATAAAAGTTTGAGGTGTTTAACTATGCATGACGGATATCCCTGTTT 8460
          ************************************************************

M-82      AAGGAGAGGTGGACTAATCTGAAATATTTCTAGATTAAGAACAGATACGTTGTGTTATGA 8519
TY172     AAGGAGAGGTGGACTAATCTGAAATATTTCTAGATTAAGAACAGATACGTTGTGTTATGA 8520
          ************************************************************

M-82      TGGGCAGCTGGTGATATTGTTATCTCTGCGTGTGCCTTTTTTTAGGCCTTTCTACCAATT 8579
TY172     TGGGCAGCTGGTGATATTGTTATCTCTGCGTGTGCCTTTTTTTAGGCCTTTCTACCAATT 8580
          ************************************************************

M-82      GAATGCTGAAAATTCATTTGCAACCAACCTTGGTTGAATTTATTTTCAGCAAGGATCCGT 8639
TY172     GAATGCTGAAAATTCATTTGCAACCAACCTTGGTTGAATTTATTTTCAGCAAGGATCCGT 8640
          ************************************************************

M-82      CATAATTCTCAGATATCCAATCATCAACAACTTTGAACTTCTATTAAATTTTAGGGCATC 8699
TY172     CATAATTCTCAGATATCCAATCATCAACAACTTTGAACTTCTATTAAATTTTAGGGCATC 8700
          ************************************************************

M-82      TCAGTTGTGTTCTTTCATCTTTTGATTTTATATGCAATTTTACTGTAGTAATAGGAGTAT 8759
TY172     TCAGTTGTGTTCTTTCATCTTTTGATTTTATATGCAATTTTACTGTAGTAATAGGAGTAT 8760
          ************************************************************

M-82      CTCTCTTCAATGCTTTGGCCATATCGGTACGAGAGAATTTATCATCTGATGTGCCCTCTC 8819
TY172     CTCTCTTCAATGCTTTGGCCATATCGGTACGAGAGAATTTATCATCTGATGTGCCCTCTC 8820
          ************************************************************

M-82      CTTTACTTTTTTGCAGACAGAAAATACGATAAGGAAGTCTTAATTAAAAATATGCGTGTG 8879
TY172     CTTTACTTTTTTGCAGACAGAAAATACGATAAGGAAGTCTTAATTAAAAATATGCGTGTG 8880
          ************************************************************

M-82      CTTGATTTTCTGGTTTTAAGGATATTTAGGTCTAAAAACTATAGTTACATTACATAATTT 8939
TY172     CTTGATTTTCTGGTTTTAAGGATATTTAGGTCTAAAAACTATAGTTACATTACATAATTT 8940
          ************************************************************

M-82      AGGGATGCTAGATGTAGAGGTCTTTTGCTAGAGCGAGTGCTTGTTTGAACCCCCCGCCCC 8999
TY172     AGGGATGCTAGATGTAGAGGTCTTTTGCTAGAGCGAGTGCTTGTTTGAACCCCCCGCCCC 9000
          ************************************************************
```

```
M-82       GCCCCGCATATTTCATGAATTATGATATTAATTGGATATTTATGATACCGCTTTTCAAGC 9059
TY172      GCCCCGCATATTTCATGAATTATGATATTAATTGGATATTTATGATACCGCTTTTCAAGC 9060
           ************************************************************

M-82       TTTCTGAAGAAACTAAGAAGGATAACATCTTTATTTAAAAACTTTTTTCCCTCTCTTGTT 9119
TY172      TTTCTGAAGAAACTAAGAAGGATAACATCTTTATTTAAAAACTTTTTTCCCTCTCTTGTT 9120
           ************************************************************

M-82       ATAACTTCTTGCAATAAATGTAGTCATGTCCCTTTTTCTGGATCTCTTAACATTTATATT 9179
TY172      ATAACTTCTTGCAATAAATGTAGTCATGTCCCTTTTTCTGGATCTCTTAACATTTATATT 9180
           ************************************************************

M-82       AATGAGCCCCTGCATGAAGTTTACTTCCAGTTGTCAACAAATAGATCCTTGTAGTGTGTT 9239
TY172      AATGAGCCCCTGCATGAAGTTTACTTCCAGTTGTCAACAAATAGATCCTTGTAGTGTGTT 9240
           ************************************************************

M-82       TCTTTACCCGAAACTTGTGAAAATTGAAGTTACTTTATTTGGACTTCTCTAGGATCCTGA 9299
TY172      TCTTTACCCGAAACTTGTGAAAATTGAAGTTACTTTATTTGGACTTCTCTAGGATCCTGA 9300
           ************************************************************

M-82       TCGTGCATGCTATGGACCAAAGCATGTTGAAGTTGCCCATGAGCGTCTGGCTATTCAGAC 9359
TY172      TCGTGCATGCTATGGACCAAAGCATGTTGAAGTTGCCCATGAGCGTCTGGCTATTCAGAC 9360
           ************************************************************

M-82       ACTTCTCATTACTGACGAGCTCTTTAGGTGAGTATCTTATGGTCCCAGGTTAATGGGGGC 9419
TY172      ACTTCTCATTACTGACGAGCTCTTTAGGTGAGTATCTTATGGTCCCAGGTTAATGGGGGC 9420
           ************************************************************

M-82       TTTATGAGCAATAAAATTAAACTGTATATAGCTTGATATAAATTTGCAACTCGTGGCATA 9479
TY172      TTTATGAGCAATAAAATTAAACTGTATATAGCTTGATATAAATTTGCAACTCGTGGCATA 9480
           ************************************************************

M-82       TTTCAAGTTCATAAGTATCTCTTTTTACGTGCTTAGCTTTTAAAATCGACATCTTTGGTT 9539
TY172      TTTCAAGTTCATAAGTATCTCTTTTTACGTGCTTAGCTTTTAAAATCGACATCTTTGGTT 9540
           ************************************************************

M-82       CATAGGATAATATGTAAACATGTATAACTATCTTCAAATCTCAACCAGTTATGTATGGGC 9599
TY172      CATAGGATAATATGTAAACATGTATAACTATCTTCAAATCTCAACCAGTTATGTATGGGC 9600
           ************************************************************

M-82       CATTTCACTCGTGCTGTATATATATAGTTGGTTGATATCGTACATGAGAAATATCCAACT 9659
TY172      CATTTCACTCGTGCTGTATATATATAGTTGGTTGATATCGTACATGAGAAATATCCAACT 9660
           ************************************************************

M-82       TCTTTTGATCTGTTGACAGGAGTTCTGATGTAGAAACGAGGAAAAAGTATGCTAATTTGG 9719
TY172      TCTTTTGATCTGTTGACAGGAGTTCTGATGTAGAAACGAGGAAAAAGTATGCTAATTTGG 9720
           ************************************************************

M-82       TCGATTCAGTCAAGGATTCAGGTGGTACTGCTCTCATTTTCTCGTCAATGCATGTCTCCG 9779
TY172      TCGATTCAGTCAAGGATTCAGGTGGTACTGCTCTCATTTTCTCGTCAATGCATGTCTCCG 9780
           ************************************************************

M-82       GAGAACGTGAGTATATACAATTCCTGTTAATTTCTTTTTCCTCCCAGCATTTTCATCTCT 9839
TY172      GAGAACGTGAGTATATACAATTCCTGTTAATTTCTTTTTCCTCCCAGCATTTTCATCTCT 9840
           ************************************************************

M-82       GCCTTTCCCTGTCGCCCCCTAGTAATGCTTAGAATGGTATTTTCCTTCTGTGCACATATT 9899
TY172      GCCTTTCCCTGTCGCCCCCTAGTAATGCTTAGAATGGTATTTTCCTTCTGTGCACATATT 9900
           ************************************************************
```

```
M-82            CATTGTCCCATAGTCGCTTCAAATCCTTTTTCCTTCCTACGAACACACGTTCTTTACTGC 9959
TY172           CATTGTCCCATAGTCGCTTCAAATCCTTTTTCCTTCCTACGAACACACGTTCTTTACTGC 9960
                ************************************************************

M-82            ATATTTGTCAAGGCTGATTAAAACAACTTTTGGTTTTCGTCACAGAATTGAATCAGCTAA 10019
TY172           ATATTTGTCAAGGCTGATTAAAACAACTTTTGGTTTTCGTCACAGAATTGAATCAGCTAA 10020
                ************************************************************

M-82            CCGGCATTGCTGCAATCCTTCGTTTTCCTTTGCCGGAGCTGGAAGACATTGAGATGTGAT 10079
TY172           CCGGCATTGCTGCAATCCTTCGTTTTCCTTTGCCGGAGCTGGAAGACATTGAGATGTGAT 10080
                ************************************************************

M-82            GGATGAAATGATCGTTCATCGGTAATGAATGTTATCTGCACTGTTAAAAATGTGACTATT 10139
TY172           GGATGAAATGATCGTTCATCGGTAATGAATGTTATCTGCACTGTTAAAAATGTGACTATT 10140
                ************************************************************

M-82            CACTATAAAATGAAGTGAATACACAGAAACTGTAATGTTTTTCTAGCAGTGGAAGTAACA 10199
TY172           CACTATAAAATGAAGTGAATACACAGAAACTGTAATGTTTTTCTAGCAGTGGAAGTAACA 10200
                ************************************************************

M-82            TCATCGACTGATGTTGCGTATCGCGTGTTGTATATTCGTTTTTTTTTTTTTTACTAATTG 10259
TY172           TCATCGACTGATGTTGCGTATCGCGTGTTGTATATTCGTTTTTTTTTTTTTTACTAATTG 10260
                ************************************************************

M-82            AAATGTAATATATCGTCGATTATCGAGGTGTACTATATCAGTGTAACGATATTTTTCATC 10319
TY172           AAATGTAATATATCGTCGATTATCGAGGTGTACTATATCAGTGTAACGATATTTTTCATC 10320
                ************************************************************

M-82            GAATATGTTGGCTTTTAGATAAAGTTTTCTGGTTGTTCAT 10359
TY172           GAATATGTTGGCTTTTAGATAAAGTTTTCTGGTTGTTCAT 10360
                ****************************************
```
