# Supplementary material for: A Novel Route Controlling Begomovirus Resistance by the Messenger RNA Surveillance Factor Pelota
Source: PLoS Genet. 2015 Oct 8;11(10):e1005538. doi: 10.1371/journal.pgen.1005538 (PMC4598160; doi:10.1371/journal.pgen.1005538)
Supplement: S1 Table — Markers are displayed according to their approximate distance in Kb from Nac1. (PDF) [file pgen.1005538.s007.pdf]

S1 Table. DNA markers displaying polymorphisms between the resistant line TY172 and the susceptible counterpart M-82, on tomato chromosome 4. Markers are displayed according to their approximate distance in Kb from *Nac1*.

Marker	Distance from *Nac1* (Kb)	Forward (F) and Reverse (R) primers	Restriction enzyme
Nac1	0	F- TGCCTGGTTTCTGCTGTCA<br>R- TAAAGCTGAAGAAGGACTTACCCT	*TaqI*
31	6	F- TTCCATTGACACTTGTCCTGAG<br>R- CCGTTTGTATAACGATAAGGGC	*Hinf*I
22	6.7	F- CCCTTATCGTTATACAAACGGC<br>R- TTTGTGCGAGAATTGATGAGAG	*Hinf*I
41	7.6	F- ATCTAATTCCATCCGGTGGTC<br>R- TCCATAAGCTTCCTTGTTGTTG	*Pml*I
34	8.7	F- CAGTTGCTCGTGTGTTTTGTTC<br>R- GCGCTCTATAGCTACGGTTTTG	*A*clI
25	18.5	F- ATTGCTTCTAAAGTCTGGCAGG<br>R- TTTAGCTTTAAGGGGTCGTTTG	*Tsp*RI
1.8	33.2	F- CCTTGACAATCAACAATGCTTC<br>R- TCACGTGTGTGATTTATGCTTG	*Alu*I
RP5	184.3	F- ATTCCTCTTCAATAGGTCCACG<br>R- GTCTCATCTGTTTCCGAGTTCC	*Alu*I
RP3	186.5	F- CAAACTCTCCCATGCTTCTTTC<br>R- TCATTCGGGAAATTAACCTACG	*Bgl*II
7.4	218.7	F- GTGAATTATCATGGCACCATTC<br>R- TATGAAGCTACGATATTCCGGG	*Ear*I
DJ	220.7	F- ATAGTTCAAGAAAAAGTCGAACC<br>R- CGAATTAAAATCATGAAACAATG	PCR
Ring	231.7	F- GTTCATGATGGAGTAGAAGAAGAAG<br>R- CTTAGTCAACCCTCTTTAGGTTGC	*Mnl*I
4.4	249.9	F- AAAATTTAAGGAATCCCATAGCG<br>R- TTTGAAATATCTTATGTTTGTTCGG	*Afl*III
Pelo	268.2	F- AAATTTGTTTCATTCAATATGAAGATTGT<br>R- CGTTTTCTTCATCTGGGGT	McSNP
4.8	270.7	F- GGTTCTTTTGAGCTATAACTCTGC<br>R- ATATTGGGCCAACCATAACTTC	*Rsa*I
9.4	271.6	F- AATTAATGTGAGACGGAGCCAG<br>R- CCAAGTCACATGGTAAATGCAG	*Ase*I
5.4	273.9	F- CAAATATGTCAATGGATGGTGG<br>R- AAGGTGCGGTGGATTATTAAAG	*Hph*I
3.1	291.6	F- GTAAATAAAGCACAAATGCCCC<br>R- AATTGTTGTGCCACCACTAGAC	*Cvi*KI
9.8	301.3	F- TAACGAACACCAACTGTTCGAC<br>R- ATCATTTCCCCTTACCATTCAC	*Nla*IV
0.1	316.2	F- CTAGCCTCTGTGTAAGATCGGG<br>R- TCAAAACATCTCAAACGACCTC	*Bsa*AI
0.3	333.3	F- CCAGTGATAATGATGGATGTGG<br>R- ATGGAAAAATGATGGGACTACG	*Bcc*I
0.4	340.8	F- TGCTATTGACTTTTCCACTTGC<br>R- TTGAATTTCACGTCATTGGC	*Hinf*I/*Cla*I
0.5	348.9	F- TTCATATTGATCACTTGCGGTC<br>R- ATTTGGCTAATGGAATCTCCTG	*Ava*II
5.8	351	F- CTTGTAAACCCATGTGGTAGGG<br>R- AACGTGTGAGTGAAGCAAAAAG	*Bgl*II
3.8	579.3	F- TTTTTACCTCGGGAGCTAGGAG<br>R- GCCGAGATAACATTAGTGGTGAC	*Hph*I
4.9	693	F- AGTGCCACGTAAGCTAAAAAGG<br>R- AAGTTGATCCAATTCAAGCTGC	*BstU*I
C2_At5g37360	43,552	F- AGCTCATGAATGCTTATTGTGAC<br>R- ACCATATTAAGAGTAAACTTTTACAAATG	*Taq*I
